# Capsule modulation enhances immunity and delays resistance in MDR *Acinetobacter baumannii*

**DOI:** 10.1016/j.isci.2026.115761

**Published:** 2026-04-16

**Authors:** Fangfang Shen, Yuanfei Wang, Qiong Wu, Minglong Yang, Biquan Chen, Pengfei Wu, Ying Ye

**Affiliations:** 1 Department of Infectious Diseases, The First Affiliated Hospital of Anhui Medical University, Hefei, Anhui 230027, China; 2 Department of Infectious Diseases, Anhui Provincial Children’s Hospital, Children’s Hospital of Fudan University (Affiliated Anhui Branch), Hefei, Anhui 230022, China; 3 Department of Neurosurgery, The First Affiliated Hospital of USTC, Division of Life Sciences and Medicine, University of Science and Technology of China, Hefei, Anhui 230001, China; 4 Department of Infectious Diseases, Anqing Municipal Hospital, Anqing, Anhui 246003, China; 5 Anhui Key Laboratory of Brain Function and Diseases, Hefei, Anhui 230001, China; 6 Anhui Provincial Stereotactic Neurosurgical Institute, Hefei, Anhui 230001, China; 7 Anhui Provincial Clinical Research Center for Neurosurgical Disease, Hefei, Anhui 230001, China; 8 Anhui Provincial Key Laboratory of Tumor Evolution and Intelligent Diagnosis and Treatment, Bengbu Medical University, Bengbu, Anhui 233030, China

**Keywords:** microbiology, bacteriology

## Abstract

Multidrug-resistant *Acinetobacter baumannii* (MDRAB) is a serious clinical threat. Although bacteriophages provide pathogen-specific therapy, the rapid emergence of phage resistance limits clinical durability. Here, we isolated a lytic phage, WYF231108.1, from hospital wastewater targeting the clinical isolate AHMU_SF230901.1 (SF). Through serial passage, we generated phage-resistant derivatives (SF_R1, SF_R2, SF_R3) and host-range-adapted phages (WYF_V1, WYF_V2, WYF_V3). Genomic and phenotypic profiling showed that resistance coincided with capsule attenuation and downregulation of capsule-biosynthesis genes, accompanied by reduced virulence and enhanced recognition by innate immune cells. Adapted phages restored lytic activity against resistant strains and, when combined with antibiotics, suppressed resistant outgrowth *in vitro* and improved survival in murine infection models. Collectively, our results define an evolutionary trade-off in which phage resistance associated with capsule attenuation reduces pathogenicity while exposing a therapeutic vulnerability. This vulnerability may be exploited using an adapted phage-antibiotic combination regimen.

## Highlights

• Capsule modulation enhances immune recognition and delays resistance in *A. baumannii*
• Phage-resistant mutants show reduced virulence and increased immune susceptibility
• Phage-antibiotic synergy improves survival and delays resistance in murine models

## Graphical abstract

**Figure:**
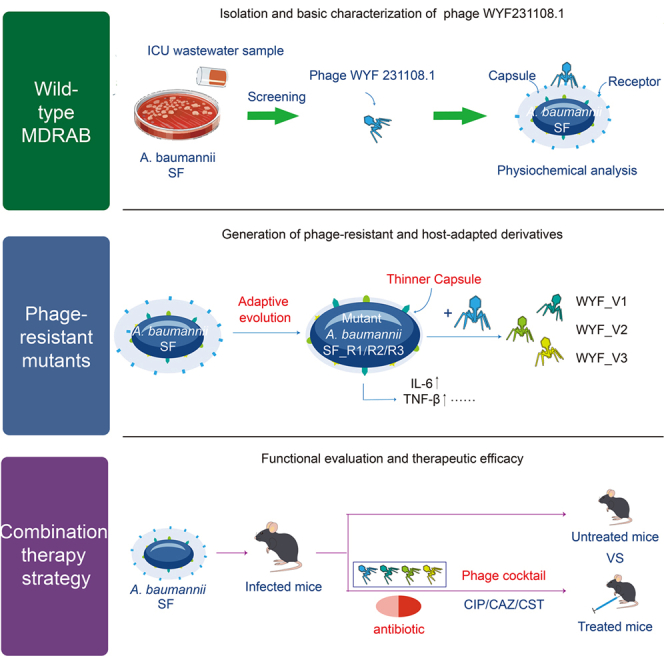

## Introduction

Multidrug-resistant *Acinetobacter baumannii* (MDRAB), particularly carbapenem-resistant strains (CRAB), is among the most challenging Gram-negative pathogens causing healthcare-associated infections worldwide, with limited treatment options available.^1^ Last-line agents (e.g., tigecycline and polymyxins) exhibit variable efficacy with dose-limiting toxicity,^2^ and the slow pace of antibiotic discovery underscores the need for alternative or adjunct anti-infective strategies.^3^

Bacteriophage therapy has regained attention for its pathogen specificity and *in situ* amplification; however, monophage therapy often yields only transient benefit because phage-resistant variants are rapidly selected.^4^ In *Acinetobacter baumannii*, resistance mechanisms include mutations or remodeling of receptor-associated structures (capsule, surface polysaccharides, and outer-membrane proteins), activation of intracellular defenses (restriction-modification and *CRISPR-Cas*), and regulation of extracellular polysaccharide matrices and biofilms. Notably, the capsule is a common adsorption determinant for lytic phages; capsule attenuation or compositional changes can confer resistance but impose fitness trade-offs, including reduced virulence, increased antibiotic susceptibility, and heightened host immune recognition.

Rationally designed phage cocktails that diversify receptor usage, life-history traits, and lytic kinetics can lower the probability of single-locus resistance and slow the emergence of resistance.^5^ Phage-encoded depolymerases can directly degrade capsules, weakening bacterial defenses, while membrane-active antibiotics may facilitate phage entry; such combinations have shown synergy *in vitro* and *in vivo*. Despite growing evidence, systematic data in MDRAB remain limited. Accordingly, we used the *Acinetobacter baumannii* phage WYF231108.1 as a representative example to determine how phage cocktails can be assembled under principles of receptor diversity, ecological complementarity, and evolutionary constraint to effectively delay resistance, and to evaluate whether resistance-associated phenotypes (e.g., capsule attenuation) can be exploited to create therapeutic windows for immune or pharmacologic interventions. Given that WYF231108.1 shares ∼95% genome identity with a previously reported phage, our focus is on leveraging resistance-associated changes rather than emphasizing phage novelty itself.^6^^,^^7^

Here, focusing on ICU-derived MDRAB isolates and their corresponding lytic phages, we assemble multi-phage cocktails^8^ and use serial evolution and time-kill assays to quantify delays in resistance emergence and reductions in resistant mutant frequency. We integrated transcriptomic and phenotypic analyses to delineate alterations in capsule-associated pathways. We further evaluate the consequences of resistance trade-offs for immune activation (cytokine release) and antibiotic susceptibility, and test the efficacy of cocktails alone or combined with antibiotics in a murine infection model.^9^ Our aim is to present a proof-of-concept framework linking receptor diversity and resistance-associated trade-offs to more durable control of MDRAB infections, while recognizing that validation across multiple clinical lineages and KL types will be required to assess generalizability.

## Results

### Isolation and basic characterization of *Acinetobacter baumannii* SF and phage WYF231108.1

**Figure 1:**
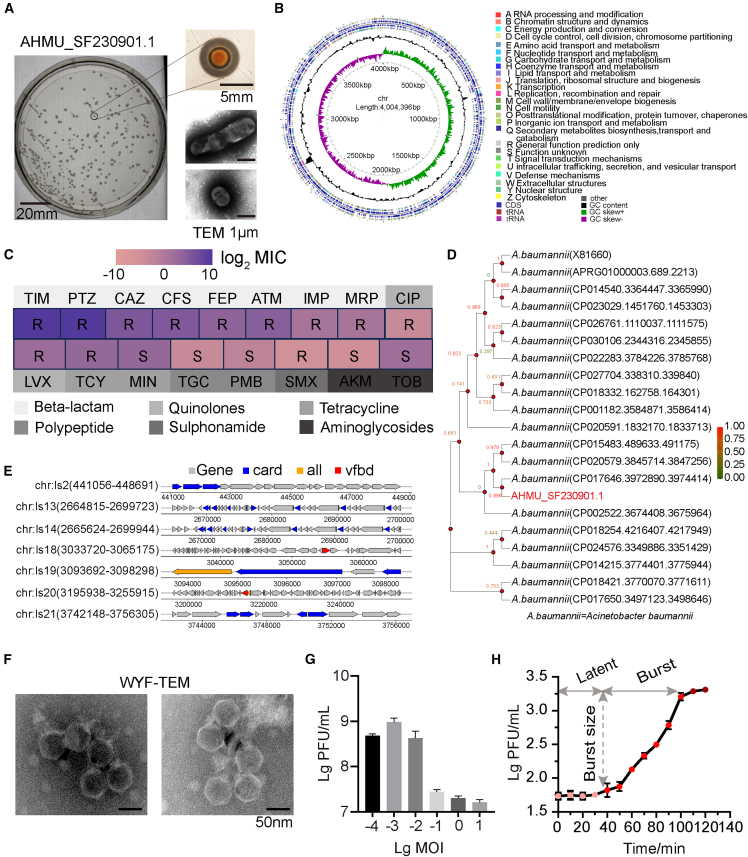
Screening and physicochemical characterization of phages targeting multidrug-resistant *Acinetobacter baumannii* (MDRAB) (A) Representative colony morphology of the clinical isolate AHMU_SF230901.1 (designated SF) on agar plates (plate diameter, 10 cm; representative colony diameter, 5 mm). The image shows a yellow colony center surrounded by a visible halo, consistent with abundant surface polysaccharide/capsular material. Right, transmission electron microscopy (TEM) images of negatively stained SF cells highlighting the capsule (electron-dense region surrounding the cells). Scale bars, as indicated (1 μm). (B) Circular map and functional annotation of the SF genome. Outer rings indicate annotated coding sequences (CDSs) colored by COG functional categories; inner rings show rRNA/tRNA features and GC content/GC skew. (C) Antimicrobial susceptibility profile of SF. Heatmap shows MIC values (log2 scale as indicated); R, resistant; I, intermediate; S, susceptible. Antibiotic abbreviations are as labeled; drug classes are indicated below the heatmap. (D) 16S rRNA-based phylogenetic analysis of SF (highlighted in red) within representative *Acinetobacter baumannii* genomes. (E) Genomic island analysis of SF. Predicted island regions are shown with annotated genes; “card” and “vfdb” denote resistance and virulence-associated hits from the CARD and VFDB databases, respectively; “all” indicates unidentifiable or multifunctional annotations. (F) Representative TEM image (negative stain) of phage WYF231108.1 particles, showing overall virion morphology. Scale bars, 50 nm. (G) Determination of optimal multiplicity of infection (MOI) for WYF231108.1. Phage yields were quantified as PFU/mL after infection at the indicated MOIs. (H) One-step growth curve of WYF231108.1 on SF. The latent period and burst phase are indicated; burst size was calculated from the increase in PFU during the rise period. Unless otherwise stated, all *in vitro* experiments were independently repeated three times; data are presented as mean ± SD.

SF was a clinical isolate obtained from routine diagnostic samples recovered in the clinical microbiology laboratory of The First Affiliated Hospital of Anhui Medical University. It formed circular colonies with a diameter of 2–5 mm on agar plates, with a distinct yellow center surrounded by a thin halo. TEM observation of negatively stained cells revealed the characteristic capsular structure of *Acinetobacter baumannii* (Figure 1A). Whole-genome sequencing showed a genome size of 4,004,396 bp (Figure 1B). Antimicrobial susceptibility testing, interpreted according to EUCAST breakpoints, indicated that SF was resistant to ticarcillin (TIM), piperacillin/tazobactam (PTZ), ceftazidime (CAZ), cefoperazone/sulbactam (CFS), cefepime (FEP), aztreonam (ATM), imipenem (IMP), meropenem (MRP), ciprofloxacin (CIP), levofloxacin (LVX), tetracycline (TCY), and sulfamethoxazole (SMX) (Figure 1C). Because SF was resistant to at least four of five tested antimicrobial classes, it met the international criteria for MDRAB. Phylogenetic analysis based on 16S rRNA showed high similarity to *Acinetobacter baumannii* ATCC 19606 (97.76% identity, 99% query coverage); meanwhile, the average nucleotide identity (ANI) with *Acinetobacter baumannii* ASM975968v1 was 97.97%, consistent with the ICSP species demarcation standard (Figure 1D). Genomic island analysis predicted the presence of multiple virulence- and resistance-associated genes (Figure 1E). Subsequently, ORFs were predicted from the assembled SF genome, and the corresponding protein sequences were extracted; BLASTp was then used to perform homology searches against the full Virulence Factor Database (VFDB) virulence factor dataset. In total, 144 ORFs were identified as VFDB hits, spanning multiple functional categories, including adhesion, immune modulation, and secretion systems (Figure S1A).

From hospital sewage, a lytic phage WYF231108.1 was isolated. TEM revealed its icosahedral head (66.4 ± 1.2 nm) and short tail (30.1 ± 3.1 nm) (Figure 1F). MOI optimization indicated that MOI = 1 × 10^−3^ yielded the highest titer (Figure 1G). The one-step growth curve suggested a latent period of approximately 40 min, followed by a 70 min rise period before reaching the plateau phase (Figure 1H). Thermal stability assays showed that infectivity was relatively stable between 25°C and 50°C, with 30 °C being optimal; treatment at 60 °C for 60 min led to near-complete inactivation, and complete loss of activity occurred at 70 °C (Figure S1B). UV irradiation reduced phage titers significantly within 10 min, and almost completely inactivated them within 30 min (Figure S1C). Phage infectivity was maintained at pH 5.0–9.0, whereas activity decreased markedly at pH 4.0 and 11.0, and was completely lost at pH 2.0 and 12.0 (Figure S1D). Exposure to 5% chloroform had little effect on phage activity (Figure S1E). Taken together, WYF231108.1 exhibits strong environmental tolerance and host specificity, suggesting potential for translational applications, particularly in combination with antibiotics for the treatment of MDRAB.^10^

### WYF231108.1-induced phage-resistant derivatives: SF_R1, SF_R2, and SF_R3

**Figure 2:**
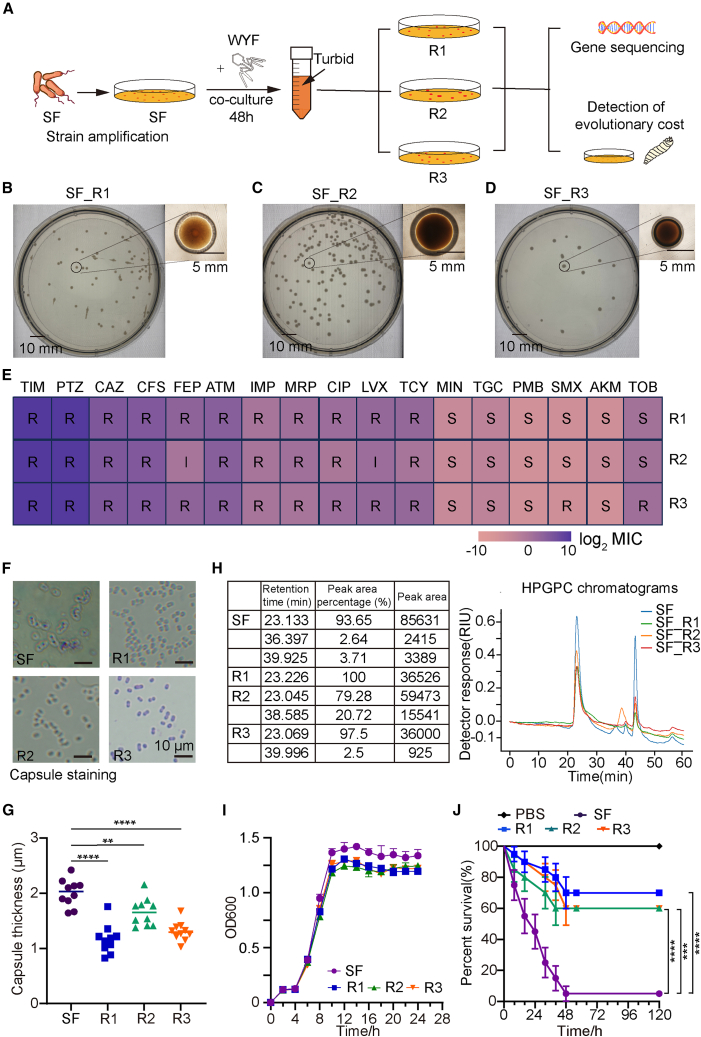
Evolutionary cost of phage-resistant MDRAB derivatives (A) Workflow for isolating phage-resistant derivatives SF_R1-SF_R3 by co-culture of SF with phage WYF231108.1 (48 h). Turbid cultures were plated, and independent resistant colonies were isolated for downstream phenotyping and genome sequencing. (B–D) Representative colony morphologies of SF_R1, SF_R2, and SF_R3 on agar plates (plate diameter, 10 cm; colony diameter, 5 mm). (E) Antimicrobial susceptibility profiles of SF_R1-SF_R3. Heatmap shows MIC values (log2 scale as indicated); R, resistant; I, intermediate; S, susceptible. (F) Representative micrographs of Anthony’s capsule staining for SF and SF_R1-SF_R3. In this staining, bacterial cells are stained, whereas the capsule appears as a clear halo surrounding each cell. Scale bars, 10 μm. (G) Quantification of capsule thickness based on Anthony-stained micrographs. Capsule thickness was measured as halo width using identical imaging settings across groups; each dot represents one cell (*n* = 10 cells per strain). Statistical significance is indicated in the figure. (H) Capsule polysaccharide extracts from SF and capsule variants SF_R1-SF_R3 were analyzed by high-performance gel permeation chromatography (HPGPC; size-exclusion chromatography). Chromatograms were recorded using a refractive index (RI) detector and are shown as an overlay. The table summarizes peak retention times and relative contributions within each sample (peak area percentage). (I) Growth curves of SF and SF_R1-SF_R3 in liquid culture, monitored by OD600 over time. (J) Survival of Galleria mellonella larvae following infection with SF or SF_R1-SF_R3 (Kaplan-Meier; log rank test). Unless otherwise stated, data represent mean ± SD from three independent experiments.

Iterative co-culture of SF with phage WYF231108.1 yielded three independent phage-resistant derivatives (SF_R1-R3). Colonies from these mutants displayed a reduced halo-to-colony-radius ratio, indicative of attenuated capsular or exopolysaccharide production (Figures 2A–2D). This phenomenon is consistent with previous studies showing that phage resistance in Acinetobacter baumannii frequently involves frameshifts in capsule-biosynthesis genes (e.g., gtr6), which remove branched sugars from the capsule and impair phage adsorption.^11^

Notably, certain mutants exhibited partial re-sensitization to antibiotics: For example, SF_R2 shifted from resistant (R) to intermediate (I) for FEP and LVX, and from R to susceptible (S) for SMX^12^ (Figure 2E), consistent with phage-antibiotic trade-offs and suggestive of potential therapeutic synergy. Anthony capsule staining confirmed thinner capsules in mutants SF_R1 (1.16 ± 0.30 μm), SF_R2 (1.65 ± 0.23 μm), and SF_R3 (1.30 ± 0.20 μm) compared with wild-type SF (2.00 ± 0.19 μm) (Figures 2F and 2G), a structural change likely increasing envelope permeability and altering drug-response profiles. To biochemically confirm that this effect was not caused by differences in culture conditions, we compared capsular polysaccharide (CPS) levels in *Acinetobacter baumannii* grown under identical conditions using the phenol-sulfuric acid method.^13^ The results showed that the mutants contained less polysaccharide than the SF strain (Figure S1F). In addition, high-performance gel permeation chromatography (HPGPC) indicated that, compared with SF, the phage-resistant mutants SF_R1, SF_R2, and SF_R3 exhibited reduced polysaccharide content, and SF_R2 and SF_R3 displayed an increased proportion of low-molecular-weight fractions (Figure 2H).

Functionally, phage-resistant mutants showed reduced growth and virulence relative to SF, with *G*. *mellonella* survival improved by 55–65% (Figures 2I and 2J). Genome re-sequencing mapped variants to capsule-related loci and pathways, with enrichment across carbohydrate metabolism, energy metabolism, and membrane transport (Figure S1G). These findings agree with previous observations that capsule defects both confer phage resistance and enhance immune clearance.

### Isolation and characterization of phage host-adapted derivatives WYF_V1, WYF_V2, and WYF_V3

**Figure 3:**
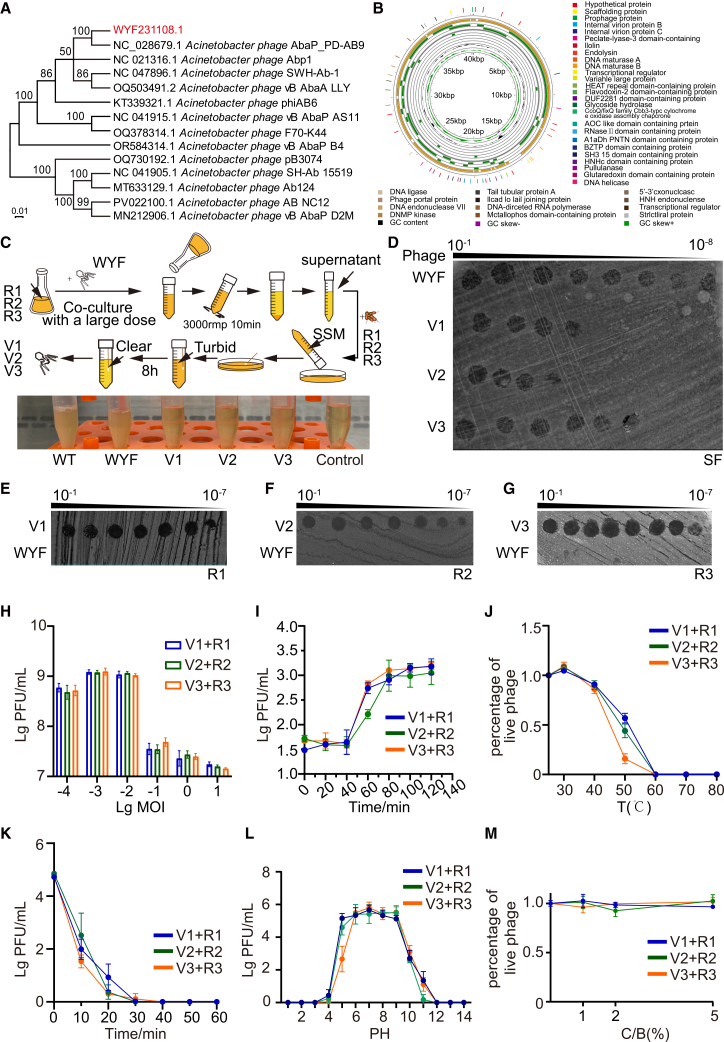
Physicochemical characterization of host-adapted phage derivatives WYF_V1-WYF_V3 (A) Phylogenetic analysis of WYF231108.1 with representative Acinetobacter phages based on large terminase subunit (TerL); WYF231108.1 is highlighted. (B) Circular genome map of WYF231108.1 shows annotated ORFs and GC content/GC skew. (C) Workflow for isolating host-adapted derivatives (WYF_V1-WYF_V3) through iterative propagation on resistant hosts (SF_R1-SF_R3). After high-dose co-culture, phage-containing supernatants were used for subsequent infection cycles until culture clearance; representative turbidity outcomes are shown. (D) Spot titration assays of WYF231108.1 and host-adapted derivatives on the parental host SF (serial dilutions 10^−1^ to 10^−8^). Representative plates are shown. (E–G) Spot titration assays of WYF231108.1 and the corresponding host-adapted derivatives on SF_R1 (E), SF_R2 (F), and SF_R3 (G), respectively. (H) Optimal MOI determination for each host-adapted derivative on its corresponding resistant host (V1 on R1, V2 on R2, V3 on R3), quantified as phage yield (PFU/mL). (I) One-step growth curves of WYF_V1-WYF_V3 on their corresponding resistant hosts. (J–M) Abiotic stability of WYF_V1-WYF_V3 under the indicated conditions, including thermal stability (J), UV sensitivity (K), pH stability (L), and chloroform tolerance (M). Unless otherwise stated, all experiments were independently repeated three times; data are shown as mean ± SD.

Maximum-likelihood phylogenetic analysis based on TerL grouped WYF231108.1 with *Acinetobacter* phage AbaP_PD-AB9 in a distinct clade supported by bootstrap values > 70% (Figure 3A). Pairwise BLAST comparisons showed 95.13% nucleotide identity with 94% query coverage. Under the International Committee on Taxonomy of Viruses (ICTV) taxonomy, WYF231108.1 is assigned to the subfamily *Beijerinckvirinae* (family *Autographiviridae*, order *Caudovirales*). Although the ANI with AbaP_PD-AB9 was 94.17%, tree topology indicated that WYF231108.1 remains divergent, forming a separate branch rather than clustering within the same lineage.^14^

The WYF231108.1 genome is 40,225 bp with 39.52% GC content and 93.14% coding density (37,464 bp), comprising 47 predicted ORFs.^15^ The longest ORF (3,099 bp) encodes a putative DNA-ejection protein; several ORFs were annotated as hypothetical. No tRNA genes, antibiotic-resistance genes, or virulence factors were detected. Functional annotation indicated modules for DNA replication/modification, transcriptional regulation, lysis (endolysin/holin), DNA packaging (terminase large subunit), and structural proteins^16^ (Figure 3B).

Iterative host-phage co-culture yielded host-adapted derivatives of WYF231108.1 that regained infectivity against resistant hosts SF_R1-R3 (Figure 3C). Spot assays suggested altered infectivity profiles on the parental and resistant hosts, consistent with mutations affecting tail fibers or other receptor-binding components^17^ (Figures 3D–3G). To identify candidate genetic determinants underlying the restored host range, we sequenced and compared the receptor-binding protein loci of the three adapted phages (WYF_V1-V3), particularly the long tail fiber genes. The results showed that all adapted phages (WYF_V1-V3) harbored mutations in the long tail fiber protein. Specifically, the long tail fiber of WYF_V1 contained three amino acid substitutions (S586F, A676T, and N679D), WYF_V2 carried four nonsynonymous mutations (I599F, T617P, G634D, and A674S), and WYF_V3 exhibited three amino acid substitutions (Q592H, A595S, and A615T) (Figure S1H). While optimal MOI was comparable to the wild-type phage, the host-adapted derivatives exhibited lower replication efficiency and narrower abiotic tolerance windows, with reduced pH stability, UV tolerance, and thermal robustness, whereas chloroform sensitivity was unchanged^18^^,^^19^ (Figures 3H–3M). These performance trade-offs reflect adaptive evolution under host selection and align with the phage-bacterium arms-race paradigm.^20^

### Phage-antibiotic synergy and resistance delay mediated by WYF231108.1 and host-adapted derivatives

**Figure 4:**
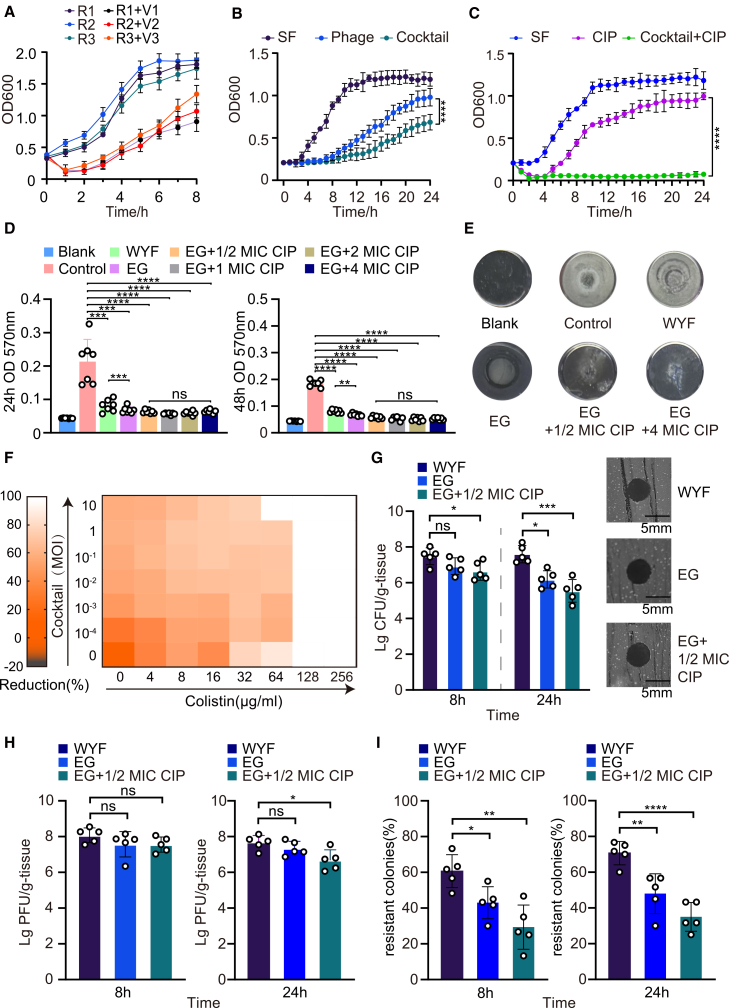
Phage-host coevolution and phage-antibiotic combinations delay resistance and enhance efficacy (A) Growth dynamics (OD600) of resistant derivatives (SF_R1-SF_R3) in the presence of the corresponding host-adapted phage derivatives (V1-V3), compared with untreated controls. (B) Growth inhibition of SF treated with WYF231108.1 monotherapy versus the phage cocktail (as defined in the main text), monitored by OD600. (C) Growth inhibition of SF under ciprofloxacin (CIP) alone versus phage cocktail +1× MIC CIP, monitored by OD600. (D) Crystal violet biofilm biomass assay. Biofilms formed under the indicated treatment conditions were stained with crystal violet; bound dye was solubilized and quantified by absorbance at 570 nm (OD570). Measurements are shown at 24 h and 48 h. (E) Representative microscopic images of biofilms formed under the indicated conditions and time points, showing treatment-associated differences in biofilm architecture consistent with the quantitative crystal violet results. Scale bars, as indicated. (F) Checkerboard combination assay of the phage cocktail with CIP. Heatmap shows growth reduction (%) across phage MOI-antibiotic concentration matrices. (G) *In vivo* bacterial burdens (CFU per g tissue) under the indicated treatment regimens at 8 h and 24 h post-infection; representative plaque assays are shown on the right. Each dot represents one mouse; bars indicate mean ± SD. (H) *In vivo* phage titers (PFU per g tissue) under the indicated treatment regimens at 8 h and 24 h post-infection. (I) Frequency of resistant colonies recovered from infected mice at 8 h and 24 h post-infection under the indicated treatments. (A–E) show biological replicates (*n* = 3). For (F–I), individual data points are shown with mean ± SD; statistical analyses were performed as indicated in the figure (two-tailed Student’s *t* test or multiple-comparison analysis; ∗*p* < 0.05, ∗∗*p* < 0.01, and ∗∗∗*p* < 0.001; ns, not significant).

Host-adapted derivatives WYF_V1-V3 effectively inhibited the growth of their corresponding phage-resistant hosts (Figure 4A). A four-phage cocktail comprising WYF231108.1 and WYF_V1-V3 (EG group) delayed the time for cultures to reach OD600 = 0.6 by > 9 h compared with single-phage WYF231108.1 (Figure 4B), consistent with complementary receptor usage and broader target coverage. When combined with CIP at 1× MIC (CIP), the cocktail further suppressed MDRAB growth^21^ (Figure 4C). Biofilm-disruption assays showed that, at 48 h, the phage-antibiotic combination, EG+ 1/2 MIC CIP, EG+ 1 MIC CIP, EG+2 MIC CIP, and EG+4 MIC CIP, outperformed single-phage treatment (Figures 4D–4F). Meanwhile, to determine whether this effect could reduce CIP usage while maintaining antibacterial efficacy, we compared 1× MIC CIP alone with a “phage cocktail +1/2 MIC CIP” regimen; no significant difference in inhibitory efficacy was observed (Figures S1I and S1J). We also examined colistin and a β-lactam antibiotic (CAZ); this synergistic effect remained, supporting the potential generalizability of this approach, subject to isolate- and capsule type-dependent validation (Figures S1K and S1L).

To establish the bacteremia model, mice were intraperitoneally injected with 500 μL of bacterial suspensions corresponding to 2 × 10^5^, 2 × 10^6^, or 2 × 10^7^ CFU per mouse (i.e., 4 × 10^5^, 4 × 10^6^, or 4 × 10^7^ CFU/mL, respectively). The 4 × 10^7^ CFU/mL dose (2 × 10^7^ CFU/mouse) caused 100% mortality within 24 h and was defined as LD100, whereas all mice survived the 4 × 10^6^ CFU/mL dose (2 × 10^6^ CFU/mouse). Therefore, we used 5 × 10^6^ CFU per mouse (i.e., 1 × 10^7^ CFU/mL in a 500 μL inoculum) as the challenge dose for bacterial-burden, resistance-frequency, and survival experiments. In the murine bacteremia model, both the phage cocktail treatment and the cocktail combined with antibiotic treatment groups reduced the bacterial burden within 8 h compared with the single-phage treatment group, with the combination therapy showing the strongest bactericidal effect. At 24 h, the other two groups showed significantly lower bacterial burdens compared with the single-phage treatment group (Figure 4G). In addition, by monitoring the *in vivo* persistence of phages after administration, we found that phages persisted *in vivo* over the observation period, suggesting a sustained therapeutic effect (Figure 4H). The cocktail-plus-antibiotic regimen significantly lowered the frequency of emergent resistant colonies (Figure 4I). Together, these results indicate that phage-antibiotic synergy enhances bacterial clearance while delaying resistance development.

### Enhanced host immune recognition and clearance of phage-resistant SF_R1-SF_R3

**Figure 5:**
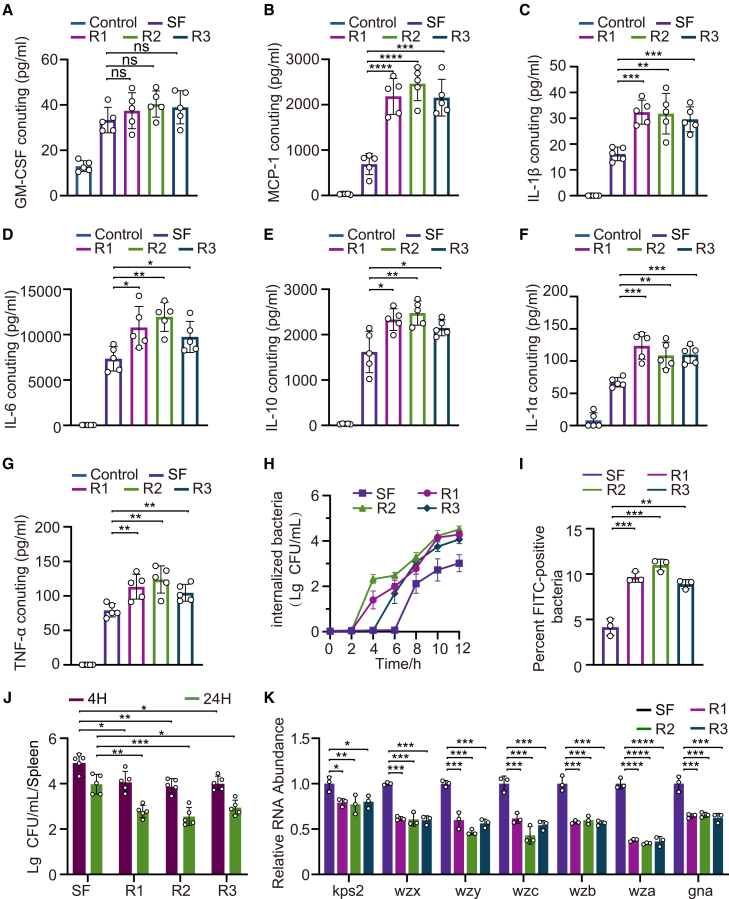
Capsule attenuation in phage-resistant derivatives promotes innate immune clearance (A–G) Cytokine and chemokine responses of murine peritoneal macrophages stimulated with formalin-fixed bacteria (SF or SF_R1-SF_R3). Supernatants were analyzed using a 21-plex Luminex assay (detection limit <10 pg/mL); representative readouts are shown (including GM-CSF, MCP-1, IL-1β, IL-6, IL-10, IL-1α, and TNF-α). Each dot represents an independent replicate; bars indicate mean ± SD. (H) Kinetics of bacterial uptake by RAW264.7 macrophages incubated with FITC-labeled SF or SF_R1-SF_R3; intracellular bacteria were quantified over time (flow cytometry-based internalization readout). (I) Flow cytometric quantification of FITC-positive macrophages after co-incubation with FITC-labeled bacteria (10,000 events acquired; singlet gating). Percent FITC-positive cells is shown as mean ± SD. (J) Competitive co-infection assay *in vivo*. Mice were co-injected with equal CFU of SF and a mutant strain (total inoculum 5 × 10^6^ CFU); splenic bacterial loads of each competitor were quantified at 4 h and 24 h post-infection. (K) Relative expression of capsule biosynthesis genes in SF versus SF_R1-SF_R3 measured by RT-qPCR (normalized to the SF control and an internal reference gene). Statistical significance is indicated (∗*p* < 0.05, ∗∗*p* < 0.01, and ∗∗∗*p* < 0.001). All panels represent three independent experiments unless otherwise stated; data are shown as mean ± SD.

CPSs facilitate immune evasion; therefore, capsule-thinned mutants are more readily recognized and phagocytosed by murine peritoneal macrophages. Consistent with increased innate immune susceptibility, serum complement-mediated killing assays showed that SF_R1-R3 exhibited significantly reduced survival in active serum compared with SF, whereas no significant difference was observed in heat-inactivated serum, indicating enhanced sensitivity to complement-dependent killing (Figure S2A). Similarly, in the PMN killing assay, SF_R1-R3 displayed markedly lower survival than SF, and the survival difference was further amplified in the presence of serum (Figure S2B), supporting increased susceptibility to neutrophil-mediated clearance. Multiplex cytokine and chemokine assays showed that, compared with the wild-type SF, the average level of GM-CSF was slightly increased but not significantly different (Figure 5A), whereas levels of IL-6, IL-10, TNF-α, IL-1α, IL-1β, and MCP-1 were significantly elevated in response to the mutants, indicating stronger pathogen-associated molecular pattern (PAMP)-dependent activation^22^ (Figures 5B–5G). Phagocytosis assays and flow cytometry confirmed that the mutants were engulfed earlier and more efficiently (Figures 5H and 5I). *In vivo*, splenic CFU counts demonstrated more rapid clearance of the mutants (Figure 5J). Consistently, the expression of capsule biosynthesis-related genes (such as *kps2*, *wzx,* and *wzy*) was downregulated in phage-resistant derivatives^8^^,^^23^^,^^24^^,^^25^^,^^26^ (Figure 5K). Collectively, capsule attenuation exposes innate immune ligands, thereby enhancing macrophage recognition and bacterial clearance.

### Efficacy of the WYF231108.1 + WYF_V1 + WYF_V2 + WYF_V3 phage cocktail in a murine infection model

**Figure 6:**
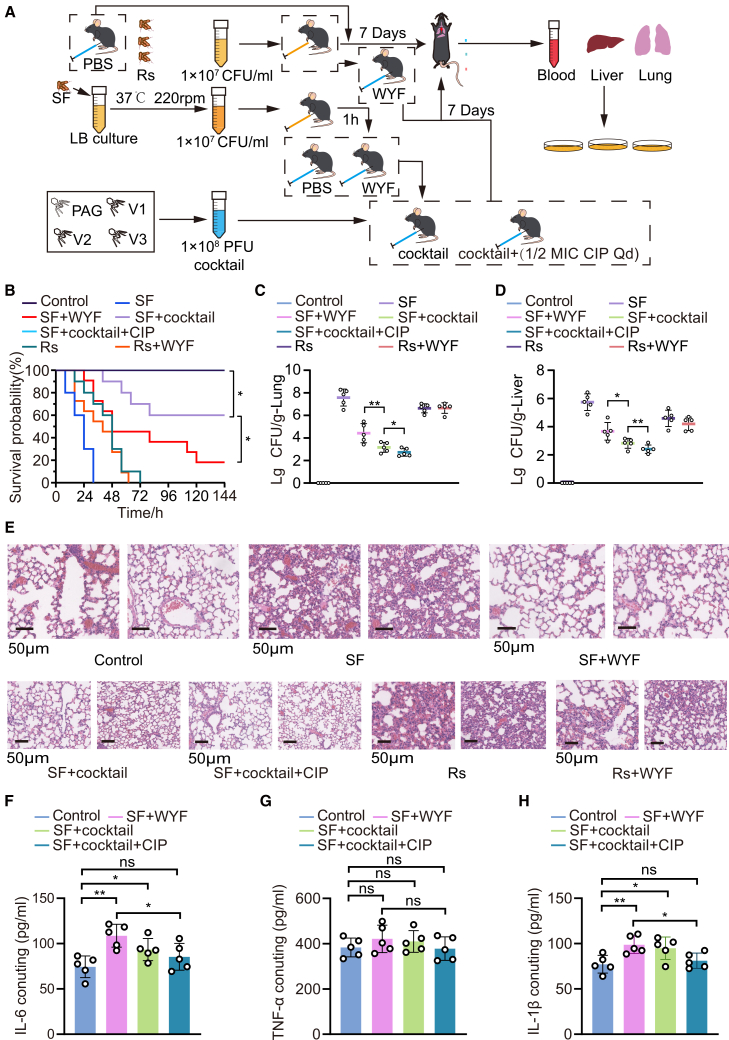
*In vivo* efficacy of phage cocktail-antibiotic therapy and associated inflammatory responses (A) Schematic of the murine bacteremia model and treatment regimen. Mice were intraperitoneally injected with SF (1 × 10^7^ CFU/mL; 500 μL; i.e., 5 × 10^6^ CFU per mouse) or resistant derivatives (Rs), with PBS as a control. Treatments were administered at the indicated time points, including single-phage WYF231108.1, the phage cocktail, or phage cocktail plus sub-MIC ciprofloxacin (1/2 MIC CIP); sample collection is indicated. (B) Kaplan-Meier survival analysis of infected mice under the indicated treatments (log rank test; significance annotated). (C and D) Residual bacterial burdens in lung (C) and liver (D) at 24 h post-infection (CFU/g tissue). Each dot represents one mouse; bars indicate mean ± SD. (E) Representative lung histopathology sections (H&E staining) from the indicated treatment groups at 7 days post-treatment. H&E images were used to assess tissue architecture and inflammation-associated injury; injury severity was evaluated based on the extent of alveolar destruction and inflammatory cell infiltration. Scale bars, 50 μm. (F–H) Serum inflammatory cytokines were measured at the indicated endpoint (e.g., IL-6, TNF-α, and IL-1β). Statistical analyses were performed as indicated (two-tailed Student’s *t* test or multiple comparisons; ∗*p* < 0.05, ∗∗*p* < 0.01, ∗∗∗*p* < 0.001, and ∗∗∗∗*p* < 0.0001; ns, not significant). Survival experiments were performed independently using n = 10 mice per group. For all other in vivo experiments, unless otherwise stated, each data point represents an individual mouse.; data are shown as mean ± SD.

Phage cocktails combined with antibiotics may enhance macrophage-mediated clearance through the mechanism of capsule attenuation. Compared with phage monotherapy or antibiotic monotherapy, the combination regimen increased survival by approximately 40% (Figures 6A and 6B) and significantly reduced residual bacterial loads in the lungs and liver (Figures 6C and 6D). Histopathological examination showed that lung alveolar damage was alleviated after combination therapy, whereas the wild-type or mutant-only bacterial groups exhibited pronounced inflammation (Figure 6E). Serum analyses showed that, compared with the WYF monotherapy group, the combination regimen elicited a stronger early proinflammatory cytokine response (TNF-α, IL-6, and IL-1β) (Figures 6F–6H). This heightened inflammatory response was consistent with more effective pathogen clearance, as reflected by a faster reduction in bacterial burden; notably, by day 7, these cytokines returned to near-baseline levels, suggesting that the combination enhanced clearance without causing sustained inflammation, consistent with synergistic immune modulation. To evaluate translational feasibility, we further monitored anti-phage humoral responses after dosing; ELISA measurements at day 10 and day 21 showed increased anti-phage IgG (and detectable IgM/IgA) signals in the active cocktail group compared with buffer or inactivated cocktail controls (Figures S2C–S2E). In addition, serum biochemistry parameters (ALP, AST, BUN, and creatinine) and H&E histology of major organs (liver, spleen, and kidney) did not reveal overt toxicity or tissue injury in the cocktail-alone (EG) compared with controls (Figures S2F–S2L). Taken together, the “phage-mutant-antibiotic” strategy not only enhances bacterial clearance but also helps suppress the emergence of resistance.^27^^,^^28^

## Discussion

This study interrogates the evolutionary trade-offs that connect phage resistance, the bacterial capsule, host immunity, and combination therapy, and yields two principal insights. First, using a clinically anchored MDRAB isolate (SF) with matched phages, phage-resistant derivatives, and host-range-adapted (infectivity-restored) phages, we show that under phage selection, bacteria commonly attenuate CPS expression to acquire resistance.^29^^,^^30^^,^^31^ This adaptation carries an immunological fitness cost: Increased exposure of PAMPs renders the bacteria more susceptible to recognition and clearance by macrophages.^32^ Second, building on this framework, a cocktail comprising wild-type and adapted phages, combined with low-dose CIP, enhanced antibacterial activity and delayed resistance *in vitro*, while reducing tissue bacterial burdens and improving survival in murine infection models.

Mechanistically, CPS are dual-purpose: They are major determinants for the adsorption of many *Acinetobacter baumannii* phages and, simultaneously, critical barriers to host innate immunity. In our experiments, capsule attenuation dampened phage adsorption via capsule-dependent receptors, yet simultaneously increased recognition by phagocytes and complement, accelerating innate responses and elevating pro-inflammatory cytokines and chemokines; together these effects improved bacterial clearance.^33^^,^^34^ The concurrent increase in inflammatory readouts provides a quantitative correlate of the resistance cost. Under phage selection pressure, bacteria reduce aspects of virulence to evade phage killing, thereby creating a therapeutic window in which host immunity can more effectively clear infection.^35^

Therapeutically, the tri-component regimen (“wild-type phages + adapted phages + antibiotic”) integrates three layers of synergy: (1) Target synergy: phages with distinct receptor binding profiles cover multiple entry routes, reducing escape via single-receptor variation^27^; (2) Evolutionary synergy: when resistance arises through capsule remodeling, adapted phages can reutilize or reprogram adsorption to reinfect resistant clones, narrowing the adaptive window^36^^,^^37^; (3) Pharmacological synergy: capsule attenuation and altered envelope permeability facilitate antibiotic penetration and killing, enabling sub-inhibitory CIP to contribute meaningfully to therapy.

Collectively, these results indicate that our strategy yields translatable *in vivo* benefits while being supported by evolutionary and immunological mechanisms, as reflected in the following three points: First, *in vivo*, these synergies translated into lower bacterial loads in the lung and liver and improved survival, supporting the relevance of the *in vitro* antibacterial effects. Importantly, WYF231108.1 itself is highly similar (∼95% genome identity) to a phage that has already been reported, so the new part of our work is how we use this phage within a resistance-capsule-immunity framework and a simple combination regimen, rather than the phage discovery itself.^38^^,^^39^ Second, It strategically deploys adapted phages with restored infectivity in a validated sequence, in which initial resistance emergence is followed by phage adaptation and a delayed onset of secondary resistance, all in a clinically relevant context.^40^ Third, it functionally substantiates the immune mechanism linking capsule attenuation to increased immune susceptibility through phagocytic kinetics and cytokine profiling, going beyond phenotypic observation alone.^41^

Future work should therefore (1) integrate phage genome engineering with receptor mapping to pinpoint structural determinants of infectivity restoration and (2) broaden immunologic profiling to include neutrophils, complement activity, and adaptive immune responses (e.g., anti-phage antibodies and repeat-dosing clearance kinetics).^42^

In sum, we propose and demonstrate in a proof-of-concept SF system a precision phage strategy that exploits resistance-associated trade-offs. While the design principle may be transferable, the phage selection/cocktail composition is expected to be isolate- and capsule type-dependent, and broader validation across diverse MDR*AB* isolates is warranted.

### Limitations of the study

Limitations warrant consideration. Host range: Most validation used a single clinical MDRAB isolate and its derivatives; multicenter, genomically diverse cohorts are needed for generalization.^43^ Molecular mechanisms: While we confirm the capsule-thinning-immune-susceptibility axis functionally, the phage mutations that restore infectivity and the decisive nodes within capsule biosynthesis pathways remain to be precisely mapped. Safety: Although efficacy and preliminary safety were demonstrated, dose-exposure-response relationships, anti-phage antibody dynamics, and clearance kinetics under repeat dosing require systematic evaluation. For potential clinical repeat dosing, anti-phage antibody levels may need to be monitored longitudinally. Strategies such as reformulating or rotating phage combinations, optimizing dosing intervals, and considering local delivery routes could help mitigate immune clearance and preserve efficacy. Regimen optimization: The combinatorial space across antibiotic classes/doses, timing, and routes of administration should be explored to balance resistance delay, toxicity reduction, and efficacy. Because *Acinetobacter baumannii* exhibits substantial KL-type heterogeneity and phage-host matching is receptor-dependent, our findings from SF should be interpreted as proof-of-concept rather than evidence of pan-strain universality. Future studies should evaluate a panel of clinical isolates spanning major KL types and use matched capsule-targeting phages to test the reproducibility of the “resistance → capsule attenuation → therapeutic window” axis.

## Resource availability

### Lead contact

Requests for further information and resources should be directed to and will be fulfilled by the lead contact, Pengfei Wu (wupengfei@ustc.edu.cn).

### Materials availability

This study generated phage-resistant bacterial derivatives and host-adapted phage derivatives, which are available from the lead contact upon request.

### Data and code availability

#### Data

NCBI Assembly: ASM975968v1 was used as the reference assembly for comparative genomic analysis. The complete genome sequence of phage WYF231108.1 has been deposited in GenBank: PX097700. Any additional information required to reanalyze the data reported in this paper is available from the lead contact, Pengfei Wu (wupengfei@ustc.edu.cn), upon request.

#### Code

This study does not report original code.

#### Additional information

Any additional information or materials required to reanalyze the data reported in this paper are available from the lead contact upon request.

## Acknowledgments

We are very grateful to all individuals and groups involved in this study. This study was supported by the 10.13039/501100001809National Natural Science Foundation of China (no.: 82573367 and 82202868), the 10.13039/501100002858China Postdoctoral Science Foundation (no.: 2022TQ0326, no.: 2023M733391), the 10.13039/501100003995Natural Science Foundation of Anhui Province (no.: 2208085QH251), 2022 Anhui Province Postdoctoral Researchers’ Scientific Research Activity Funding Project (no.: 2022B580), and the Open Project of Anhui Provincial Key Laboratory of Tumor Evolution and Intelligent Diagnosis and Treatment (no.: KFKT202401).

## Author contributions

F.S., Y.W., and Q.W. contributed equally to this work. Y.Y. and B.C. conceived the platform and led the project. P.W. and Y.Y. gathered funding and provided guidance with experimental design. F.S., Y.W., and Q.W. performed all experiments. Y.W., F.S., and Q.W. co-wrote the paper. Y.W., M.Y., P.W., and Y.Y. designed the animal experiments and analyzed the results. M.Y., B.C., and P.W. contributed to data analysis and provided advice on this work.

## Declaration of interests

The authors declare no competing interests.

## STAR★Methods

### Key resources table

**Table:** 

REAGENT or RESOURCE	SOURCE	IDENTIFIER
**Bacterial and virus strains**		

*Acinetobacter baumannii* AHMU_SF230901.1 (SF)	This paper	ASM975968v1
Phage WYF231108.1	This paper	GenBank: PX097700
Phage WYF_V1	This paper	N/A
Phage WYF_V2	This paper	N/A
Phage WYF_V3	This paper	N/A
Phage-resistant strain SF_R1	This paper	N/A
Phage-resistant strain SF_R2	This paper	N/A
Phage-resistant strain SF_R3	This paper	N/A

**Biological samples**		

Human polymorphonuclear neutrophils (PMNs)	STEMCELL Technologies	Cat# 70025

**Chemicals, peptides, and recombinant proteins**		

Triton X-100	Sigma-Aldrich	Cat# T8787
Crystal violet	Sigma-Aldrich	Cat# C0775

**Critical commercial assays**		

Mouse IL-6 ELISA Kit	Elabscience	Cat# E-EL-M0044c
Mouse IL-10 ELISA Kit	Elabscience	Cat# E-EL-M0049c
Mouse TNF-α ELISA Kit	Elabscience	Cat# E-EL-M0048c
Mouse IL-1α ELISA Kit	Elabscience	Cat# E-EL-M0029c
Mouse IL-1β ELISA Kit	Elabscience	Cat# E-EL-M0037c
Mouse MCP-1 (CCL2) ELISA Kit	Elabscience	Cat# E-EL-M0006c
Mouse PAMP ELISA Kit	MLBIO	Cat# ml093386
PCR Purification Kit	Beyotime Biotechnology	Cat# D0033
TIANamp Virus DNA/RNA Kit	TIANGEN Biotech	Cat# DP315

**Deposited data**		

A. baumannii genome sequence	NCBI	ASM975968v1
Phage WYF231108.1 genome	GenBank	PX097700

**Experimental models: Cell lines**		

RAW264.7 macrophage cells	Procell	Cat# CL-0190
Galleria mellonella larvae	Shanghai Gogo Biological	N/A
C57BL/6 mice (female, 6–8 weeks old)	Experimental Animal Center, Anhui Medical University	N/A

**Experimental models: Organisms/strains**		

Galleria mellonella larvae	Shanghai Gogo Biological	N/A

**Oligonucleotides**		

qPCR primers	This paper	See Method details

**Software and algorithms**		

GraphPad Prism	GraphPad Software	RRID:SCR_002798
Easyfig	Sullivan et al.^44^	RRID:SCR_000667
MEGA software	Kumar et al.^34^	RRID:SCR_000131
SPAdes genome assembler	Bankevich et al.^45^	RRID:SCR_010835
RAST annotation server	RAST	RRID:SCR_018206
tRNAscan-SE	Lowe Lab	RRID:SCR_015810
VFDB database	VFDB	http://www.mgc.ac.cn/VFs; RRID:SCR_001575
CARD database	CARD	https://card.mcmaster.ca; RRID:SCR_011209
Enrichr	Ma’ayan Lab	https://maayanlab.cloud/Enrichr; RRID:SCR_006483
ImageJ	ImageJ	https://ImageJ.net/; RRID: SCR_003070
NIS-Elements D	Nikon Corporation	RRID:SCR_014329
FlowJo	FlowJo LLC	RRID:SCR_008520
BeadView	BD Biosciences	RRID:SCR_018036

**Other**		

Luria-Bertani (LB) broth	BD Biosciences	Cat# 244620
Luria-Bertani agar	BD Biosciences	Cat# 244520
SM buffer	Standard laboratory reagent	N/A
0.22 μm sterile syringe filter	Biosharp	N/A
DNase I	Thermo Fisher Scientific	Cat# EN0521
RPMI-1640 medium	Gibco	Cat# 11875093
Illumina sequencing platform	Sangon Biotech	N/A
ChemiDoc MP Imaging System	Bio-Rad	Cat# 17001402
Tecnai T12 transmission electron microscope	FEI Company	Tecnai T12
VITEK 2 system	bioMérieux	AST-XN05 card
QuantStudio Real-Time PCR System	Applied Biosystems	QuantStudio 7

### Experimental model and study participant details

#### Microbial strains and bacteriophage

The clinical isolate AHMU_SF230901.1 (SF) of *Acinetobacter baumannii* was obtained from a blood culture sample of a hospitalized patient. Bacterial strains were routinely cultured in Luria–Bertani (LB) broth at 37 °C with shaking at 220 rpm unless otherwise specified.

The lytic bacteriophage WYF231108.1 was isolated from hospital wastewater using SF as the host strain. Phage-resistant derivatives (SF_R1, SF_R2, SF_R3) were generated through serial phage challenge, and host-range adapted phages (WYF_V1, WYF_V2, WYF_V3) were obtained through serial passage on resistant bacterial strains.

#### Cell lines

The murine macrophage cell line RAW264.7 was obtained from Procell (China) and maintained under standard cell culture conditions.

#### Primary cell cultures

Murine peritoneal macrophages were isolated from healthy mice by peritoneal lavage and used for cytokine and chemokine production assays following bacterial stimulation.

Human polymorphonuclear neutrophils (PMNs) were purchased as cryopreserved cells from STEMCELL Technologies and used for bacterial killing assays according to the manufacturer’s instructions.

#### Animal models

Six-to eight-week-old female C57BL/6 mice were used for infection experiments. All animal experiments were approved by the Experimental Animal Ethics Committee of the First Affiliated Hospital of the University of Science and Technology of China (Anhui Provincial Hospital), and we have complied with all relevant ethical regulations. For all rodent housing rooms, mice were maintained on a 12 h light/12 h dark cycle, with lights on at 6 a.m. and lights off at 6 p.m. Temperature range is kept between 20°C and 24°C, and humidity range is 30–70%.

*Galleria mellonella* larvae weighing approximately 150–200 mg were used as an invertebrate infection model. Larvae were maintained in the dark at 37 °C prior to infection experiments.

#### Ethics approval

This study protocol was approved by the Experimental Animal Ethics Committee of the First Affiliated Hospital of the University of Science and Technology of China (Anhui Provincial Hospital) (NO. 2025-N(A)-0150). Histopathological assessments were performed by pathologists.

### Method details

#### Bacterial strains and growth conditions

A clinical isolate of Acinetobacter baumannii, designated AHMU_SF230901.1, was obtained from the Clinical Microbiology Laboratory of the First Affiliated Hospital of Anhui Medical University (Hefei, China). A single colony was then inoculated into 5 mL lysogeny broth (LB) and grown at 37 °C with shaking at 220 rpm for 8 h. Species identification was performed by PCR amplification of the 16S rRNA gene using universal bacterial primers 27F and 1492R (Sangon Biotech, China), followed by Sanger sequencing and BLAST analysis against the NCBI database. For long-term storage, cultures were mixed with sterile glycerol to a final concentration of 20% (v/v) and stored at −80°C.

#### Phage isolation and purification

Wastewater samples were collected from the ICU wastewater station of the Second Affiliated Hospital of Anhui Medical University, centrifuged (5,000 × g, 10 min, room temperature) to remove debris, and passed through 0.22 μm sterile syringe filters (Biosharp, China) to eliminate bacterial cells. The clinical isolate *Acinetobacter baumannii* (SF) was grown to mid-log phase (OD_600_ ≈ 0.6; 1 × 10^8^ CFU/mL). For enrichment, 10 mL of the wastewater filtrate was mixed with 10 mL of LB broth and inoculated with 200 μL of the SF culture. After overnight incubation at 37 °C, the mixture was centrifuged again (5,000 × g, 10 min) and filtered through a 0.22 μm sterile filter to obtain a crude phage lysate. The presence of phages was assessed using the double-layer agar method with SF as the host, where plaque formation indicated lytic activity. Well-isolated plaques were picked and re-plated by the double-layer agar method for at least three successive rounds until morphologically uniform phage plaques were obtained. Phage genome alignment (Easyfig) was performed using Easyfig 2.2.3^44^. Finally, evolutionary relationship analysis was conducted with MEGA 11 to determine the phylogenetic relationships of phages based on the large terminase subunit.

#### Species identification by 16S rRNA gene sequencing

The nearly full-length 16S rRNA gene was amplified using universal primers 27F (5′-AGAGTTTGATCMTGGCTCAG-3′) and 1492R (also referred to as PABR1492; 5′-TACGGYTACCTTGTTACGACTT-3′). Each 25 μL PCR contained 3 μL genomic DNA (≈30 ng), 1.5 μL of each primer (15 pmol), 12.5 μL of 2× Master Mix, and 6.5 μL nuclease-free water. Thermocycling was: 95 °C for 5 min; 30 cycles of 94 °C for 1 min, 58 °C for 1 min, and 72 °C for 30 s; followed by a final extension at 72 °C for 10 min. Amplicons were resolved on 1% agarose gels and visualized under UV transillumination using a GeneRuler DNA ladder (50 bp/1 kb).

#### Purification and sequencing of PCR product

The 16S rRNA amplicon was purified using a PCR Purification Kit (Beyotime Biotechnology, China) according to the manufacturer’s instructions. Purified products were subjected to bidirectional Sanger sequencing with primers 27F and 1492R. Sequencing was performed on an ABI 373A automated DNA sequencer (Applied Biosystems, USA). Raw chromatograms were quality-trimmed and assembled into a consensus sequence using BioEdit v7.2.5. Taxonomic assignment was conducted with BLASTN against the NCBI nucleotide (nt) database; the top-scoring hit (highest identity and coverage) was used to determine species-level identity.

#### Antimicrobial resistance of *Acinetobacter baumannii* isolate

Antimicrobial susceptibility was assessed for ticarcillin; piperacillin/tazobactam; ceftazidime; cefoperazone/sulbactam; cefepime; aztreonam; imipenem; meropenem; ciprofloxacin; levofloxacin; tetracycline; minocycline; tigecycline; polymyxin B; sulfamethoxazole; tobramycin; and amikacin. Minimum inhibitory concentrations (MICs) were determined with the VITEK 2 automated system using the AST-XN05 card and interpreted according to European Committee on Antimicrobial Susceptibility Testing (EUCAST) breakpoints.

#### Whole-genome resequencing of phage-resistant mutants

Genomic DNA from the parental strain and phage-resistant mutants was extracted from overnight cultures using a bacterial genomic DNA extraction kit according to the manufacturer’s instructions. DNA quality and concentration were assessed using a NanoDrop spectrophotometer, Qubit fluorometer, and agarose gel electrophoresis. Sequencing library construction and Illumina sequencing were performed by Personal Biotechnology Co., Ltd. (Shanghai, China). Raw sequencing data were subjected to quality control and preliminary bioinformatics analysis by the sequencing provider. Clean reads were mapped to the complete genome sequence of the parental strain, which served as the reference genome, for variant detection. Identified single-nucleotide polymorphisms (SNPs) and small insertions/deletions (InDels) were filtered to remove low-quality and low-confidence variants. Retained variants were annotated based on the reference genome annotation and further categorized according to their predicted biological functions, with particular attention to genes associated with capsule biosynthesis, cell surface structures, membrane-associated processes, and phage resistance.

#### HPGPC analysis of polysaccharide molecular weight

The molecular weight and size distribution of polysaccharide samples were determined by high-performance gel permeation chromatography (HPGPC) using a Waters 1515 HPLC system equipped with a Waters 2414 refractive index (RI) detector and a Waters 2707 autosampler. Two aqueous SEC columns (Shodex Ohpak SB-804 HQ and SB-806M HQ, 8 × 300 mm) were connected in series, with 0.05 M NaCl as the mobile phase (filtered through a 0.22 μm membrane and ultrasonically degassed for 20 min) at a flow rate of 0.50 mL/min, a column temperature of 40 °C, and an injection volume of 30 μL. Molecular weight calibration was performed using carbohydrate standards including glucose, maltotetraose, dextran, and pullulan (180-1,060,000 Da), and a calibration curve was generated by plotting log(MW) versus elution volume (V) to calculate sample molecular weights. Samples were prepared at 5 mg/mL (5 mg polysaccharide dissolved in 1 mL 0.05 M NaCl), filtered through a 0.22 μm membrane, and then injected for analysis.

#### Comparative genomic analysis of the tail fiber gene

The long tail fiber (receptor-binding protein) ORF was identified in the assembled genomes, and the corresponding regions of WYF_V1-V3 were aligned to WYF231108.1 (BLASTN-based comparative genomics) to call nonsynonymous substitutions; amino acid changes were mapped to the tail fiber protein to generate the schematic.

#### Transmission electron microscopy

Phage morphology was examined by TEM using a concentrated preparation of WYF231108.1 (≈1 × 10^11^ PFU/mL). A drop of the suspension was applied to carbon-coated copper grids, allowed to adsorb, and excess liquid was wicked off with filter paper. Grids were negatively stained with 2% (w/v) phosphotungstic acid for 5 min, air-dried, and imaged on a Tecnai T12 transmission electron microscope (FEI, USA) operated at 120 kV.

#### Optimal multiplicity of infection

To determine the optimal multiplicity of infection (MOI; ratio of phage particles to bacterial cells) for phage amplification, mid-log *Acinetobacter baumannii* SF (≈1 × 10^8^ CFU/mL) was mixed with phage WYF231108.1 at MOIs of 1, 0.1, 0.01, 0.001, and 0.0001. Phage suspensions were prepared by serial 10-fold dilution in SM buffer, and for each condition 100 μL of bacteria was combined with 100 μL of phage. Following a 10 min adsorption at room temperature, mixtures were transferred into 5 mL LB and incubated at 37 °C with shaking (220 rpm) until lysis (4–6 h) or overnight. Crude lysates were clarified (10,000 × g, 10 min), and phage titers (PFU/mL) were quantified by the double-layer agar method. The MOI that yielded the highest final titer was defined as the optimal MOI.

#### One-step growth curve

Host bacteria (*Acinetobacter baumannii* SF) were infected with phage WYF231108.1 at the predetermined optimal MOI and allowed to adsorb for 15 min at 37 °C. Cells were pelleted (12,000 × g, 10 min), the supernatant was discarded to remove unbound phage, and the pellet was washed twice with LB. The washed cells were resuspended in 20 mL LB and incubated at 37 °C with shaking (220 rpm). Aliquots were collected every 10 min for up to 120 min, immediately serially diluted, and titrated by the double-layer agar method to determine PFU/mL. The latent period was defined as the interval from the end of adsorption to the onset of the rise phase, and the burst size was calculated as the ratio of the PFU increase during the rise period to the number of infected cells at time zero.

#### Thermal and pH stability

For thermal stability, aliquots of phage WYF231108.1 suspension (1 mL each) were dispensed into 1.5-mL microtubes (*n* = 3 per condition) and incubated in water baths at 25, 30, 50, 60, 70, or 80 °C for 60 min. An untreated aliquot held at 4 °C served as a control. After incubation, tubes were immediately chilled on ice, and residual infectivity was quantified as PFU/mL using the double-layer agar plaque assay.

For pH stability, LB broth was adjusted to pH 1–14 using 1 M H_2_SO_4_ or 1 M NaOH. To each pH-adjusted tube, 500 μL of phage suspension (1 × 10^5^ PFU/mL) was added and incubated at 37 °C for 60 min. Samples were then neutralized to pH 7 with acid or base as appropriate, passed through 0.22 μm sterile syringe filters, and titrated by the double-layer agar method. All conditions were tested in triplicate.

#### UV stability

For UV stability, aliquots of phage suspensions (WYF231108.1, WYF_V1, WYF_V2, and WYF_V3) in PBS were exposed to UV-C irradiation (254 nm) at a distance of 25 cm for 0, 10, 20, 30, 40, 50, or 60 min. After treatment, samples were immediately protected from light and placed on ice. Residual infectivity was quantified by the double-layer agar plaque assay and expressed as PFU/mL (shown as log10 PFU/mL). All assays were independently performed in triplicate.

#### Chloroform sensitivity assay

Chloroform tolerance of phage WYF231108.1 was evaluated by incubating aliquots of phage suspension (1 × 10^5^ PFU/mL in SM buffer) with chloroform at final concentrations of 0%, 1%, 2%, or 5% (v/v) at 37 °C for 30 min (*n* = 3 per condition). After incubation, tubes were briefly vortexed and centrifuged to separate phases; the aqueous phase was carefully recovered, aerated for 5-10 min to dissipate residual chloroform, and immediately titrated by the double-layer agar plaque assay to determine PFU/mL.

#### Anthony’s capsule staining for *Acinetobacter baumannii*

A clean glass slide was used to prepare a thin bacterial smear of *Acinetobacter baumannii* under aseptic conditions. To preserve capsules, the smear was air-dried without heat fixation. The smear was flooded with 3% crystal violet for 2–3 min and the excess stain was drained. Without rinsing with water, 20% (w/v) aqueous copper sulfate was applied for 30-60 s to decolorize the capsule and serve as a counterstain; excess copper sulfate was gently blotted off (do not wash with water). Slides were air-dried and examined under oil immersion (1,000 ×) on a microscope (Olympus, Japan). Capsules were visualized as clear halos surrounding deep-purple cells against a pale blue background.

#### Determination of host-cell lysis activity

To assess lytic activity over time, mid-log MDRAB cultures grown in LB were mixed with phage WYF231108.1 at the predetermined optimal MOI. Mixtures, along with a bacteria-only control, were incubated at 37 °C with shaking (220 rpm). At 0–12 h (hourly), 1 mL aliquots were removed, serially 10-fold diluted, and plated on LB agar for CFU enumeration after incubation. Lytic activity was expressed as the reduction in CFU relative to the bacteria-only control. All experiments were performed in triplicate.

#### Biofilm formation assay

Overnight *Acinetobacter baumannii* cultures were adjusted to ≈1 × 10^9^ CFU/mL in LB medium. Aliquots (100 μL) were dispensed into flat-bottom 96-well polystyrene plates (sterile), with LB-only wells as blanks. Plates were incubated statically at 37 °C for 24 h.

Planktonic growth was quantified by carefully transferring 100 μL supernatant from each well to a fresh microplate and reading OD_600_ on an Infinite M200 reader (Tecan, Switzerland). For biofilm measurement, the original plate was gently washed 3× with 200 μL sterile PBS to remove non-adherent cells, air-dried at room temperature (≈20–30 min), and stained with 1% (w/v) crystal violet (100 μL/well, 15 min). Excess dye was discarded and wells were rinsed 3× with distilled water, then air-dried. Bound dye was solubilized with 95% ethanol (150–200 μL/well, 10 min) and absorbance was recorded at OD_570_. Background (blank) values were subtracted. Experiments were performed in triplicate.

#### Phylogenetic analysis

The amino acid sequences of the large terminase subunit (TerL) were extracted from the assembled genome of WYF231108.1 and from representative Autographiviridae phages retrieved from NCBI. Multiple sequence alignments were performed using MUSCLE. Phylogenetic trees were reconstructed using the maximum-likelihood (ML) method in MEGA 11 with 1,000 bootstrap replicates.

#### *Galleria mellonella* infection model

Prior to experimentation, *G*. *mellonella* larvae were starved for 24 h at 37 °C in the dark in 90 mm Petri dishes. Healthy, active larvae (150-200 mg; no visible melanization) were surface-sterilized with 75% (v/v) ethanol. Using a 10 μL microsyringe (Shanghai Gaoge Industry and Trade Co., Ltd.), 10 μL of MDRAB suspension (1 × 10^8^ CFU/mL; dose = 1 × 10^6^ CFU/larva) was injected into the right proleg of the last abdominal segment. A control group received 10 μL sterile PBS.

To assess phage therapy, 30 min after bacterial challenge, phage WYF231108.1 diluted in SM buffer to achieve an MOI of 0.001 relative to the inoculum was administered (10 μL/larva) via the same injection site; a vehicle control group received 10 μL SM buffer alone. Larvae were maintained at 37 °C in darkness and survival was recorded every 8 h for 120 h. Death was defined as lack of response to gentle probing with a syringe needle. Experiments were performed in biological replicates, with group sizes provided in the figure legends.

#### Production of cytokines and chemokines by *Acinetobacter baumannii*-stimulated peritoneal macrophages

Peritoneal lavage cells were harvested from naive (uninfected) mice. Cell suspensions were adjusted to 5 × 10^5^ cells/mL and seeded at 1 mL per well into 24-well tissue-culture plates (Beyotime Biotechnology, China). Adherent peritoneal macrophages were then stimulated with 5 × 10^7^ formalin-fixed *Acinetobacter baumannii* cells per well-either the parental SF strain (ffSF) or its phage-resistant derivatives (ffSF_R1, ffSF_R2, ffSF_R3); medium alone served as a negative control. Supernatants were collected at 24 h and 48 h after stimulation, clarified by centrifugation, and stored at −20°C until cytokine and chemokine quantification.

#### Serum complement-mediated killing assay

Overnight cultures were subcultured 1:100 into fresh LB and grown to mid-log phase. Bacteria were washed and resuspended in PBS to 1 × 10^6^ CFU/mL. Normal serum (active serum) was prepared from naive mice and heat-inactivated serum was generated by incubation at 56 °C for 30 min. Bacterial suspensions were mixed with active or heat-inactivated serum at a final serum concentration of 20% and incubated at 37 °C for 60 min with gentle agitation. At time 0 and the indicated time points, aliquots were serially diluted and plated on LB agar for CFU enumeration. Bacterial survival was calculated relative to the initial inoculum (time 0) and presented as log CFU/mL.

#### Polymorphonuclear neutrophil killing assay

Cryopreserved human peripheral blood neutrophils (PMNs) purchased from STEMCELL Technologies were used. Vials were rapidly thawed in a 37°C water bath with gentle agitation (approximately 1–2 min; removed when a small ice crystal remained), wiped with 75% ethanol, and opened briefly in a biosafety cabinet to release pressure. The cell suspension was transferred to a 50 mL conical tube and diluted stepwise with pre-warmed RPMI 1640 (with 10% FBS or a low-serum formulation as required) to minimize DMSO exposure (first rinsing the vial with ∼1 mL medium and adding it dropwise to the cells, followed by dropwise addition of 15–20 mL medium). Cells were pelleted at 300 × g for 10 min at room temperature, the supernatant was discarded, and cells were resuspended in RPMI 1640; DNase I was added if needed to reduce clumping. Cells were counted and used immediately for downstream assays. Acinetobacter baumannii was grown overnight, washed with PBS, and the inoculum was calibrated by CFU plating. For opsonization, bacteria were incubated with either commercial complement-active serum or heat-inactivated serum (HIS) at a final concentration of 25% (v/v) at 37 °C for 30 min; HIS was prepared by heating the same serum at 56 °C for 30 min. After opsonization, bacteria were washed with PBS to remove unbound serum components. Opsonized bacteria (active-serum or HIS-treated) were mixed with PMNs at bacteria-to-PMN ratios of 10:1 and 100:1 and incubated at 37 °C with 5% CO_2_, and samples were collected at 30 min. Reactions were stopped on ice, washed to remove non-associated and non-phagocytosed bacteria, and PMNs were lysed with 1% Triton X-100 in PBS. Lysates were serially diluted, plated on TSA or blood agar, and incubated for CFU enumeration, with differences in CFU between complement-active and heat-inactivated conditions used to assess complement-dependent PMN bactericidal activity.

#### Phagocytosis assay using FITC-labeled bacteria

FITC-labeled Acinetobacter baumannii was prepared by incubating the bacteria with FITC (Sigma-Aldrich) for 30 min at 37°C in the dark, followed by washing and centrifugation to remove excess dye. Murine macrophages (RAW 264.7) or human neutrophils were cultured overnight in RPMI-1640 medium supplemented with 10% FBS and 1% penicillin-streptomycin. The cells were then incubated with FITC-labeled bacteria (MOI = 10:1) for 1 h at 37°C. After the incubation, cells were washed with PBS to remove non-phagocytosed bacteria and chilled on ice to stop phagocytosis. The cells were then analyzed by flow cytometry (BD FACSVerse, BD Biosciences) to quantify phagocytosis by measuring the percentage of FITC-positive cells and the fluorescence intensity, which correlates with the amount of internalized bacteria. Data were analyzed using FlowJo software (Tree Star).

#### Complete genome sequencing and bioinformatics analysis

Genomic DNA of phage WYF231108.1 was extracted from high-titer lysates using the TIANamp Viral DNA/RNA Kit (Tiangen Biotech, China) according to the manufacturer’s instructions. Whole-genome sequencing was performed on the Illumina platform (Sangon Biotech Co., Ltd., China). *De novo* assembly was carried out with SPAdes^45^, and the resulting consensus genome was polished by read mapping (default parameters, unless otherwise specified). Open reading frames (ORFs) were annotated using Rapid Annotation Subsystem Technology (RAST) v2.0. tRNA genes were predicted with tRNAscan-SE. Putative virulence factors and antibiotic resistance genes were screened against the Virulence Factor Database (VFDB) and the Comprehensive Antibiotic Resistance Database (CARD). A circular genome map was generated with the CGView Server. For comparative genomics, the WYF231108.1 genome was aligned to related phage genomes using BLASTN.

#### RT-qPCR

Total bacterial RNA was extracted with the M5 EASYspin Plus kit (Mei5bio, Beijing, China) and quantified using a NanoDrop spectrophotometer (Thermo Scientific, USA). Genomic DNA was removed and first-strand cDNA was synthesized with the PrimeScript RT reagent Kit with gDNA Eraser (Takara, China) following the manufacturer’s instructions (typically 500 ng-1 μg RNA per 20 μL reaction). Quantitative PCR was performed with PowerUp SYBR Green Master Mix (Applied Biosystems, Thermo Fisher Scientific, Carlsbad, USA) on a QuantStudio 7 Real-Time PCR System. Unless otherwise specified, each 20 μL reaction contained 1× SYBR mix, 0.3 μM of each primer, and 1–2 μL of diluted cDNA. Cycling conditions were: 50 °C for 2 min, 95 °C for 2 min, then 40 cycles of 95 °C for 15 s and 60 °C for 30 s, followed by a melt-curve analysis to confirm amplicon specificity. Primer efficiency (target and reference) was verified to be 90–110% using standard curves. Relative mRNA levels were normalized to 16S rRNA and expressed relative to SF using the 2^−ΔΔCt^ method. All reactions were run in technical triplicates, with at least three independent biological replicates.

#### RNA-seq data analysis

We reanalyzed a previously generated RNA-seq dataset profiling the innate immune response to Acinetobacter baumannii infection. Differential expression results from the original pipeline were used to rank significantly upregulated genes (adjusted *p* < 0.05, Benjamini-Hochberg). The top 100 upregulated DEGs (by absolute log_2_ fold change) were submitted to Enrichr (https://maayanlab.cloud/Enrichr/) for Kyoto Encyclopedia of Genes and Genomes (KEGG) pathway enrichment using the appropriate species library. KEGG terms were considered significant based on adjusted *p* values (and Enrichr combined scores where indicated). Enrichment outputs were exported and visualized as bar plots in GraphPad Prism (v10.1.2).

#### Spot assay

Overnight bacterial cultures were adjusted in LB to OD_600_ ≈ 1.0. For each plate, 100 μL of the culture was mixed with 5 mL of molten soft agar (LB containing 0.4% [w/v] agar) held at 45°C–50°C, gently vortexed, and overlaid onto pre-warmed LB agar plates. After the top agar solidified, phage suspensions prepared in SM buffer (50 mM Tris-HCl, pH 7.5; 100 mM NaCl; 8 mM MgSO_4;_ 0.01% [w/v] gelatin) were spotted (5–10 μL per spot) onto the lawn. Plates were air-dried for 10 min and incubated at 30 °C for 16 h. Plaques were visualized on a ChemiDoc MP imaging system (Bio-Rad) under white-light transillumination, and images were exported and converted to grayscale using Photoshop.

#### Anti-phage antibody ELISA

Mice were administered either buffer or an active phage cocktail (1 × 10^8^ PFU per mouse) via intraperitoneal injection once daily. Blood was collected on day 10 and day 21 after dosing, allowed to clot, and centrifuged to obtain serum.

For ELISA, high-binding 96-well plates were coated overnight at 4 °C with purified phage particles. Plates were washed with PBST, blocked with skim milk, and then incubated with serially diluted serum samples. After washing, plates were incubated with HRP-conjugated secondary antibodies against mouse IgG, IgM, or IgA. Signals were developed using TMB substrate, stopped with acid, and read at 450 nm. Background signals from blank wells were subtracted, and antibody levels were reported as OD450 at a fixed serum dilution.

#### Serum biochemistry assays

Serum ALP, AST, BUN, and creatinine were quantified using Beyotime’s Amplex Red assay kits following the manufacturer’s instructions. Briefly, serum samples were obtained as described above and stored at −80°C until analysis.

#### Cytokine and chemokine quantification

Cytokines and chemokines in culture supernatants were quantified using the MILLIPLEX MAP Mouse Cytokine/Chemokine 21-plex kit (MilliporeSigma, Billerica, USA) on a Luminex 100 IS analyzer (Luminex Corporation, USA) according to the manufacturer’s instructions. Standard curves generated from kit calibrators were fitted, and analyte concentrations were calculated with BeadView software (v1.03; Upstate Biotechnology).

#### Screening for phage-resistant mutants

From mice that received phage treatment, bacterial colonies were isolated from blood, liver, kidney, and spleen. For each mouse, at least 15 colonies were randomly selected per tissue. Individual colonies were inoculated into LB medium supplemented with phage WYF231108.1 (final dose ≈ 10^6^ PFU per well) and cultured in a microplate reader at 37 °C with intermittent shaking; OD_600_ was recorded every 15 min to generate growth curves. Relative to the wild-type *Acinetobacter baumannii* SF control grown with the same phage input, colonies that displayed uninhibited growth (i.e., growth curves comparable to phage-free controls) were provisionally classified as “putative resistant.” These putative resistant isolates were streaked twice for single-colony purification, and resistance was confirmed by liquid-culture challenge and by the soft-agar overlay/spot assay (assessment of plaque formation).

#### Confocal laser scanning microscopy

RAW264.7 murine macrophages (Procell, China) were seeded on 14-mm glass coverslips (NEST, China) placed in 24-well plates to form confluent monolayers. *Acinetobacter baumannii* was labeled with pHrodo Green (Invitrogen, Thermo Fisher Scientific) by washing fresh cultures in PBS, incubating with probe solution (20 μmol/L, 30 min, room temperature), and washing twice to remove unbound dye. Cells were infected with the pHrodo-labeled bacteria at MOI = 100 for 4 h at 37 °C (5% CO_2_). Following infection, cells were rinsed with PBS and fixed in 4% paraformaldehyde for 20 min at room temperature. F-actin and nuclei were counterstained with ActinRed 555 ReadyProbes (Thermo Fisher Scientific) and DAPI (Beyotime, China), respectively, according to the manufacturers’ instructions. Coverslips were mounted with antifade medium. Imaging was performed on a confocal laser scanning microscope (Leica system), and images were analyzed with LAS AF Lite software. pHrodo fluorescence was used as a readout of bacterial internalization, and representative fields were acquired under oil immersion (≈1,000× total magnification).

#### Murine model of *Acinetobacter baumannii* infection

Mice were randomly assigned to an infection-only group or a phage-treatment group (*n* = 10 per group). Under aseptic conditions, all mice were intraperitoneally challenged with MDRAB by injecting 500 μL of bacterial suspension at a final dose of 5 × 10^6^ CFU per mouse (1 × 10^7^ CFU/mL). The phage-treatment group received an intraperitoneal dose of the phage cocktail (1 × 10^8^ PFU) 1 h after infection via the same route. Survival was monitored for 7 days.

For therapeutic comparisons, an additional cohort was randomized into seven groups (total *n* = 70): Control (PBS), *Acinetobacter baumannii*-infected (SF), SF + ciprofloxacin (SF + CIP), SF + phage cocktail (SF + cocktail), SF + phage cocktail and ciprofloxacin (SF + cocktail + CIP), phage-resistant strain (Rs), and Rs + phage WYF231108.1 (Rs + WYF231108.1). CIP was administered at 45 mg/kg via intraperitoneal injection after infection. On day 3 and 7 post-treatment, three mice per group were randomly selected and euthanized; lung tissues, liver and serum were collected for downstream analyses.

#### Histological examination of mouse organs

The spleen, lung, kidney, and liver were harvested immediately after euthanasia and fixed in 4% paraformaldehyde (PFA) for 24 h at 4 °C. Tissues were processed through graded ethanol (70%, 80%, 95%, 100%), cleared in xylene, and embedded in paraffin. Paraffin blocks were sectioned at 3 μm on a rotary microtome (Leica RM2235), mounted on glass slides, and baked at 60 °C for 1 h. Sections were deparaffinized in xylene, rehydrated through graded ethanol to water, and stained with hematoxylin and eosin (H&E) using standard procedures. Slides were then dehydrated, cleared, and coverslipped with a resin mounting medium. Morphology was evaluated on a light microscope equipped with NIS-Elements D software (Nikon, Japan).

#### Quantitative bacteriology

Lungs and spleens were aseptically harvested, weighed, and placed into sterile tubes containing ice-cold saline (1 mL per 100 mg tissue). Tissues were homogenized with sealed, aerosol-resistant probes in a Class II biosafety cabinet. Homogenates were serially 10-fold diluted in saline, and 100 μL of each dilution was spread in duplicate onto LB agar plates. Plates were incubated at 37 °C for 16–18 h, and colonies were counted to determine viable *Acinetobacter baumannii* burdens. Results were expressed as CFU per gram of tissue (CFU/g).

### Quantification and statistical analysis

Data are shown as mean ± SD; n denotes independent biological replicates (typically *n* = 3), as stated in figure legends. Individual data points are displayed where indicated. For comparisons involving >2 groups, we used one-way or two-way ANOVA (as appropriate), followed by Tukey’s (all-pairs) or Dunnett’s (vs. control) multiple-comparison tests. For experiments with multiple cytokines or multiple ratios, *p* values were adjusted using the Benjamini-Hochberg procedure where applicable. Survival curves were analyzed by Kaplan-Meier and compared with log rank (Mantel-Cox) tests. RNA-seq pathway enrichment used Enrichr KEGG libraries; significance was defined by BH-adjusted *p* < 0.05. Graphing/statistics were performed in GraphPad Prism (V10.1.2). Significance notation: ∗*p* < 0.05, ∗∗*p* < 0.01, ∗∗∗*p* < 0.001, ∗∗∗∗*p* < 0.0001. No data were excluded unless prespecified.

## References

[1] Egido J.E., Costa A.R., Aparicio-Maldonado C., Haas P.J., Brouns S.J.J. (2022). Mechanisms and clinical importance of bacteriophage resistance. FEMS Microbiol. Rev..

[2] Beamud B., Benz F., Bikard D. (2024). Going viral: The role of mobile genetic elements in bacterial immunity. Cell Host Microbe.

[3] Kim M.S., Kim Y.D., Hong S.S., Park K., Ko K.S., Myung H. (2015). Phage-encoded colanic acid-degrading enzyme permits lytic phage infection of a capsule-forming resistant mutant Escherichia coli strain. Appl. Environ. Microbiol..

[4] Wang X., Loh B., Gordillo Altamirano F., Yu Y., Hua X., Leptihn S. (2021). Colistin-phage combinations decrease antibiotic resistance in Acinetobacter baumannii via changes in envelope architecture. Emerg. Microbes Infect..

[5] Chen L.K., Kuo S.C., Chang K.C., Cheng C.C., Yu P.Y., Chang C.H., Chen T.Y., Tseng C.C. (2017). Clinical Antibiotic-resistant Acinetobacter baumannii Strains with Higher Susceptibility to Environmental Phages than Antibiotic-sensitive Strains. Sci. Rep..

[6] Gordillo Altamirano F.L., Kostoulias X., Subedi D., Korneev D., Peleg A.Y., Barr J.J. (2022). Phage-antibiotic combination is a superior treatment against Acinetobacter baumannii in a preclinical study. EBioMedicine.

[7] Cha K., Oh H.K., Jang J.Y., Jo Y., Kim W.K., Ha G.U., Ko K.S., Myung H. (2018). Characterization of Two Novel Bacteriophages Infecting Multidrug-Resistant (MDR) Acinetobacter baumannii and Evaluation of Their Therapeutic Efficacy in Vivo. Front. Microbiol..

[8] Gordillo Altamirano F., Forsyth J.H., Patwa R., Kostoulias X., Trim M., Subedi D., Archer S.K., Morris F.C., Oliveira C., Kielty L. (2021). Bacteriophage-resistant Acinetobacter baumannii are resensitized to antimicrobials. Nat. Microbiol..

[9] Bagińska N., Grygiel I., Orwat F., Harhala M.A., Jędrusiak A., Gębarowska E., Letkiewicz S., Górski A., Jończyk-Matysiak E. (2024). Stability study in selected conditions and biofilm-reducing activity of phages active against drug-resistant Acinetobacter baumannii. Sci. Rep..

[10] Mukhopadhyay S., To K.K.W., Liu Y., Bai C., Leung S.S.Y. (2023). A thermosensitive hydrogel formulation of phage and colistin combination for the management of multidrug-resistant Acinetobacter baumannii wound infections. Biomater. Sci..

[11] Timoshina O.Y., Kasimova A.A., Shneider M.M., Arbatsky N.P., Shashkov A.S., Shelenkov A.A., Mikhailova Y.V., Popova A.V., Hall R.M., Knirel Y.A., Kenyon J.J. (2023). Loss of a Branch Sugar in the Acinetobacter baumannii K3-Type Due To Frameshifts in the gtr6 Glycosyltransferase Gene Leads To Susceptibility To Phage APK37.1. Microbiol. Spectr..

[12] Lucidi M., Imperi F., Artuso I., Capecchi G., Spagnoli C., Visaggio D., Rampioni G., Leoni L., Visca P. (2024). Phage-mediated colistin resistance in Acinetobacter baumannii. Drug Resist. Updat..

[13] Masuko T., Minami A., Iwasaki N., Majima T., Nishimura S.I., Lee Y.C. (2005). Carbohydrate analysis by a phenol-sulfuric acid method in microplate format. Anal. Biochem..

[14] Wang Z., Yang X., Wang H., Wang S., Fang R., Li X., Xing J., Wu Q., Li Z., Song N. (2024). Characterization and efficacy against carbapenem-resistant Acinetobacter baumannii of a novel Friunavirus phage from sewage. Front. Cell. Infect. Microbiol..

[15] Liu Y., Wang J., Zhao R., Liu X., Dong Y., Shi W., Jiang H., Guan X. (2024). Bacterial isolation and genome analysis of a novel Klebsiella quasipneumoniae phage in southwest China's karst area. Virol. J..

[16] Zhang H., Hu X., Ma Z., Zhen X., Tong P., Zhai G., Zhang S., Zhang W. (2024). Isolation and characterization of a relatively broad-spectrum phage against Escherichia coli. Arch. Microbiol..

[17] Oyejobi G.K., Zhang X., Xiong D., Xue H., Shi M., Yang H., Wei H. (2024). Phage-Bacterial Interaction Alters Phenotypes Associated with Virulence in Acinetobacter baumannii. Viruses.

[18] Choi Y.J., Kim S., Shin M., Kim J. (2024). Synergistic Antimicrobial Effects of Phage vB_AbaSi_W9 and Antibiotics against Acinetobacter baumannii Infection. Antibiotics (Basel).

[19] Mukhopadhyay S., Zhang P., To K.K.W., Liu Y., Bai C., Leung S.S.Y. (2023). Sequential treatment effects on phage-antibiotic synergistic application against multi-drug-resistant Acinetobacter baumannii. Int. J. Antimicrob. Agents.

[20] Gao M., Wang C., Qiang X., Liu H., Li P., Pei G., Zhang X., Mi Z., Huang Y., Tong Y., Bai C. (2020). Isolation and Characterization of a Novel Bacteriophage Infecting Carbapenem-Resistant Klebsiella pneumoniae. Curr. Microbiol..

[21] Blasco L., Ambroa A., Lopez M., Fernandez-Garcia L., Bleriot I., Trastoy R., Ramos-Vivas J., Coenye T., Fernandez-Cuenca F., Vila J. (2019). Combined Use of the Ab105-2φΔCI Lytic Mutant Phage and Different Antibiotics in Clinical Isolates of Multi-Resistant Acinetobacter baumannii. Microorganisms.

[22] Hsieh Y.C., Wang S.H., Chen Y.Y., Lin T.L., Shie S.S., Huang C.T., Lee C.H., Chen Y.C., Quyen T.L.T., Pan Y.J. (2020). Association of capsular types with carbapenem resistance, disease severity, and mortality in Acinetobacter baumannii. Emerg. Microbes Infect..

[23] Bjånes E., Koh T., Qayum T., Zurich R., McCabe S., Hampel K., Cartwright L., Nizet V. (2023). Exploring Roles of the Polysaccharide Capsule in Pathogenesis of Hypervirulent Acinetobacter baumannii Clinical Isolate Lac-4. Antibiotics.

[24] Geisinger E., Isberg R.R. (2015). Antibiotic Modulation of Capsular Exopolysaccharide and Virulence in Acinetobacter baumannii. PLoS Pathog..

[25] Rakovitsky N., Lellouche J., Ben David D., Frenk S., Elmalih P., Weber G., Kon H., Schwartz D., Wolfhart L., Temkin E., Carmeli Y. (2021). Increased Capsule Thickness and Hypermotility Are Traits of Carbapenem-Resistant *Acinetobacter baumannii* ST3 Strains Causing Fulminant Infection. Open Forum Infect. Dis..

[26] Shadan A., Pathak A., Ma Y., Pathania R., Singh R.P. (2023). Deciphering the virulence factors, regulation, and immune response to Acinetobacter baumannii infection. Front. Cell. Infect. Microbiol..

[27] Yuan Y., Wang L., Li X., Tan D., Cong C., Xu Y. (2019). Efficacy of a phage cocktail in controlling phage resistance development in multidrug resistant Acinetobacter baumannii. Virus Res..

[28] Grygorcewicz B., Wojciuk B., Roszak M., Łubowska N., Błażejczak P., Jursa-Kulesza J., Rakoczy R., Masiuk H., Dołęgowska B. (2021). Environmental Phage-Based Cocktail and Antibiotic Combination Effects on *Acinetobacter baumannii* Biofilm in a Human Urine Model. Microb. Drug Resist..

[29] Wang R., You X., Liu X., Fei B., Li Y., Wang D., Zhu R., Li Y. (2024). Characterization of phage HZY2308 against Acinetobacter baumannii and identification of phage-resistant bacteria. Virol. J..

[30] Wintachai P., Naknaen A., Pomwised R., Voravuthikunchai S.P., Smith D.R. (2019). Isolation and characterization of Siphoviridae phage infecting extensively drug-resistant Acinetobacter baumannii and evaluation of therapeutic efficacy in vitro and in vivo. J. Med. Microbiol..

[31] Roszkowiak J., Jajor P., Guła G., Gubernator J., Żak A., Drulis-Kawa Z., Augustyniak D. (2019). Interspecies Outer Membrane Vesicles (OMVs) Modulate the Sensitivity of Pathogenic Bacteria and Pathogenic Yeasts to Cationic Peptides and Serum Complement. Int. J. Mol. Sci..

[32] Rao S., Betancourt-Garcia M., Kare-Opaneye Y.O., Swierczewski B.E., Bennett J.W., Horne B., Fackler J., Suazo Hernandez L.P., Brownstein M.J. (2022). Critically Ill Patient with Multidrug-Resistant Acinetobacter baumannii Respiratory Infection Successfully Treated with Intravenous and Nebulized Bacteriophage Therapy. Antimicrob. Agents Chemother..

[33] García-Quintanilla M., Pulido M.R., López-Rojas R., Pachón J., McConnell M.J. (2013). Emerging therapies for multidrug resistant Acinetobacter baumannii. Trends Microbiol..

[34] Kumar M.D., Joshi M.P., Yadav D.M., Pandey M.S., Singhal D.C., Tyagi M.A., Gupta M.S., Jaya M.J., Tanwar M.S., Bargakshatriya M.R. (2025). Elucidating the Biofilm Composition of Acinetobacter baumannii: Uncovering Mechanisms of Host Immune Evasion. Int. J. Infect. Dis..

[35] Howard A., Reza N., Aston S., Woods B., Gerada A., Buchan I., Hope W., Märtson A.-G. (2024). Antimicrobial treatment imprecision: an outcome-based model to close the data-to-action loop. Lancet Infect. Dis..

[36] Wang M., Ning Y., Jiao X., Liu J., Qiao J. (2023). Bacteriophages and their derived enzymes as promising alternatives for the treatment of Acinetobacter baumannii infections. Arch. Virol..

[37] Fernández-Vázquez J.L., Hernández-González I.L., Castillo-Ramírez S., Jarillo-Quijada M.D., Gayosso-Vázquez C., Mateo-Estrada V.E., Morfín-Otero R., Rodríguez-Noriega E., Santos-Preciado J.I., Alcántar-Curiel M.D. (2023). Pandrug-resistant Acinetobacter baumannii from different clones and regions in Mexico have a similar plasmid carrying the blaOXA-72 gene. Front. Cell. Infect. Microbiol..

[38] Bai J., Raustad N., Denoncourt J., van Opijnen T., Geisinger E. (2023). Genome-wide phage susceptibility analysis in Acinetobacter baumannii reveals capsule modulation strategies that determine phage infectivity. PLoS Pathog..

[39] Kunisch F., Wagemans J., Yildirim S., Chan B., Schaudinn C., Lavigne R., Turner P., Raschke M., Trampuz A., Moreno M.G. (2024). Targeting MDR Pseudomonas aeruginosa biofilm with an evolutionary trained bacteriophage cocktail exploiting phage resistance trade-offs. Nat. Commun..

[40] Wang H., Yang Y., Xu Y., Chen Y., Zhang W., Liu T., Chen G., Wang K. (2024). Phage-based delivery systems: engineering, applications, and challenges in nanomedicines. J. Nanobiotechnology.

[41] Blasco L., Bleriot I., González de Aledo M., Fernández-García L., Pacios O., Oliveira H., López M., Ortiz-Cartagena C., Fernández-Cuenca F., Pascual Á. (2022). Development of an Anti-Acinetobacter baumannii Biofilm Phage Cocktail: Genomic Adaptation to the Host. Antimicrob. Agents Chemother..

[42] Wu Y., Garushyants S.K., van den Hurk A., Aparicio-Maldonado C., Kushwaha S.K., King C.M., Ou Y., Todeschini T.C., Clokie M.R.J., Millard A.D. (2024). Bacterial defense systems exhibit synergistic anti-phage activity. Cell Host Microbe.

[43] Durrant M.G., Bhatt A.S. (2021). Automated Prediction and Annotation of Small Open Reading Frames in Microbial Genomes. Cell Host Microbe.

[44] Sullivan M.J., Petty N.K., Beatson S.A. (2011). Easyfig: a genome comparison visualizer. Bioinformatics.

[45] Bankevich A., Nurk S., Antipov D., Gurevich A.A., Dvorkin M., Kulikov A.S., Lesin V.M., Nikolenko S.I., Pham S., Prjibelski A.D. (2012). SPAdes: a new genome assembly algorithm and its applications to single-cell sequencing. J. Comput. Biol..

