# Supplementary material for: Capsule modulation enhances immunity and delays resistance in MDR Acinetobacter baumannii
Source: iScience. 2026 Apr 16;29(6):115761. doi: 10.1016/j.isci.2026.115761 (PMC13186059; doi:10.1016/j.isci.2026.115761)
Supplement: Document S1. Figures S1 and S2 [file mmc1.pdf]

iScience, Volume *29*

Supplemental information

Capsule modulation enhances immunity and delays resistance in MDR *Acinetobacter baumannii*

Fangfang Shen, Yuanfei Wang, Qiong Wu, Minglong Yang, Biquan Chen, Pengfei Wu, and Ying Ye

Supplementary Figure 1

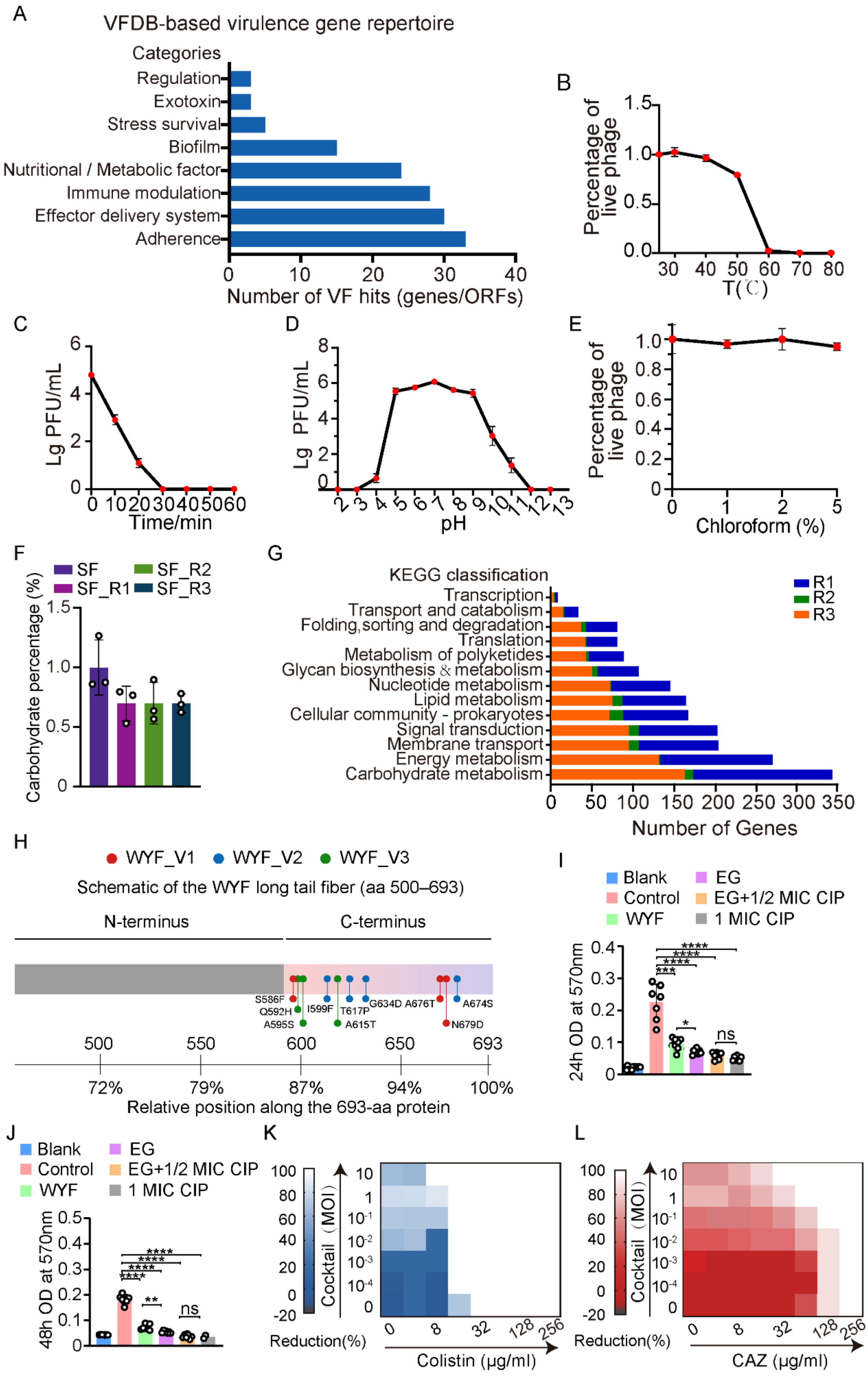

Supplementary Figure 1. Related to Figures 1, 2 and 3. (A) VFDB-based annotation of the SF genome showing the number of virulence factor hits across functional categories. The x-axis indicates the number of hit genes/ORFs, and the y-axis indicates the functional categories. (B-E) Characterization of phage WYF231108.1, including thermal stability (B), UV sensitivity (C), pH stability (D), and chloroform tolerance (E). All experiments were independently repeated three times; data are shown as mean ± SD. (F) Carbohydrate percentage of SF and capsule variants SF_R1-SF_R3. Capsule extracts were prepared under identical conditions, and the capsule extract mass is presented as a percentage relative to the parental SF. Each dot represents an independent biological replicate; bars indicate mean ± SD. (G) KEGG functional classification of evolved variants SF_R1–SF_R3. Stacked bars indicate the numbers of genes assigned to each KEGG category in SF_R1, SF_R2, and SF_R3. (H) Schematic of the WYF long tail fiber protein (693 amino acids), showing the N-terminal anchoring/assembly region and the C-terminal hypervariable region associated with receptor binding. Amino-acid substitution sites identified in the adapted phages WYF_V1 (red), WYF_V2 (blue), and WYF_V3 (green) are marked. WYF_V1 carries three substitutions (S586F, A676T, and N679D), WYF_V2 carries four nonsynonymous substitutions (I599F, T617P, G634D, and A674S), and WYF_V3 carries three substitutions (Q592H, A595S, and A615T). These variations are mainly distributed in the C-terminal region of the tail fiber. (I, J) Biofilms were formed under indicated treatment conditions and quantified by crystal violet staining followed by measuring absorbance at 570 nm (OD570). Bars indicate the mean, and dots represent independent biological replicates. (K, L) Synergistic effects of the WYF phage cocktail combined with colistin (K) or ceftazidime (L) against *A. baumannii* SF under different ratio conditions.

Supplementary Figure 2

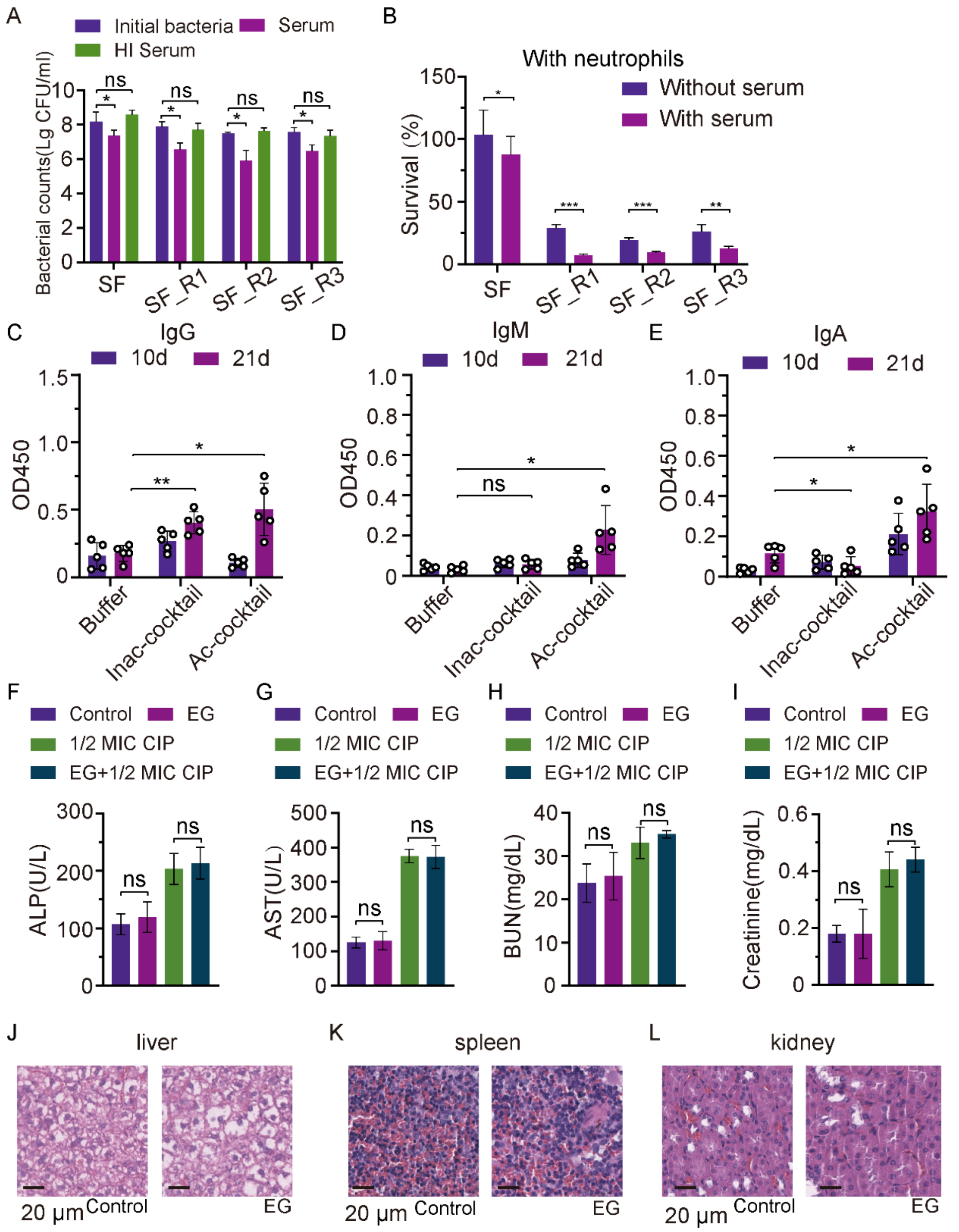

Supplementary Figure 2. Related to Figures 4, 5 and 6. (A) Serum complement-mediated killing assay of SF and SF_R1-SF_R3. After incubation in active serum or heat-inactivated serum, bacterial loads (log CFU/mL) were determined, with the initial inoculum shown as a control. (B) Polymorphonuclear neutrophil (PMN) killing assay

performed in the presence or absence of serum. Bacterial survival was calculated relative to the initial inoculum. (C-E) Anti-phage antibody responses. ELISA signals (OD450) of anti-phage IgG (C), IgM (D), and IgA (E) in mouse sera measured on days 10 and 21 after administration. Each dot represents an individual mouse, and bars indicate the group summary; statistical significance is annotated in the figure (*$P < 0.05$, **$P < 0.01$; ns, not significant). (F-I) Serum biochemical parameters across different treatment groups: ALP (F), AST (G), BUN (H), and creatinine (I); statistical significance is indicated in the figure with n = 3 mice per group; Samples were collected at day 3. (J-L) Representative H&E-stained sections of liver, spleen, and kidney from the Control and EG groups: liver (J), spleen (K), and kidney (L). Under phage-cocktail-only administration (EG), no obvious abnormalities in tissue architecture or overt inflammatory injury were observed in the liver, spleen, or kidney. Scale bar, 20 μm.
